# Methodological guidance for the evaluation and updating of clinical prediction models: a systematic review

**DOI:** 10.1186/s12874-022-01801-8

**Published:** 2022-12-12

**Authors:** M. A. E. Binuya, E. G. Engelhardt, W. Schats, M. K. Schmidt, E. W. Steyerberg

**Affiliations:** 1 Division of Molecular Pathology, the Netherlands Cancer Institute – Antoni van Leeuwenhoek Hospital, Plesmanlaan 121, 1066 CX Amsterdam, The Netherlands; 2 Department of Biomedical Data Sciences, Leiden University Medical Center, Leiden, The Netherlands; 3 Department of Clinical Genetics, Leiden University Medical Center, Leiden, The Netherlands; 4 Division of Psychosocial Research and Epidemiology, the Netherlands Cancer Institute – Antoni van Leeuwenhoek Hospital, Amsterdam, The Netherlands; 5 Scientific Information Service, The Netherlands Cancer Institute – Antoni van Leeuwenhoek Hospital, Amsterdam, The Netherlands

**Keywords:** Prediction model, Model evaluation, Validation, Impact assessment, Discrimination, Calibration, Clinical usefulness, Model updating

## Abstract

**Background:**

Clinical prediction models are often not evaluated properly in specific settings or updated, for instance, with information from new markers. These key steps are needed such that models are fit for purpose and remain relevant in the long-term. We aimed to present an overview of methodological guidance for the evaluation (i.e., validation and impact assessment) and updating of clinical prediction models.

**Methods:**

We systematically searched nine databases from January 2000 to January 2022 for articles in English with methodological recommendations for the post-derivation stages of interest. Qualitative analysis was used to summarize the 70 selected guidance papers.

**Results:**

Key aspects for validation are the assessment of statistical performance using measures for discrimination (e.g., C-statistic) and calibration (e.g., calibration-in-the-large and calibration slope). For assessing impact or usefulness in clinical decision-making, recent papers advise using decision-analytic measures (e.g., the Net Benefit) over simplistic classification measures that ignore clinical consequences (e.g., accuracy, overall Net Reclassification Index). Commonly recommended methods for model updating are recalibration (i.e., adjustment of intercept or baseline hazard and/or slope), revision (i.e., re-estimation of individual predictor effects), and extension (i.e., addition of new markers). Additional methodological guidance is needed for newer types of updating (e.g., meta-model and dynamic updating) and machine learning-based models.

**Conclusion:**

Substantial guidance was found for model evaluation and more conventional updating of regression-based models. An important development in model evaluation is the introduction of a decision-analytic framework for assessing clinical usefulness. Consensus is emerging on methods for model updating.

**Supplementary Information:**

The online version contains supplementary material available at 10.1186/s12874-022-01801-8.

## Background

Clinical prediction models are tools that predict health outcomes either at present (diagnostic) or in the future (prognostic) [1, 2]. Such models are commonly based on regression analyses of multiple variables (predictors) and increasingly on more computationally-intensive machine learning algorithms. In this era of personalized medical decision-making, models have become ubiquitous to support clinicians in predicting individualized (absolute) risk using a combination of patient and disease characteristics [2]. For example, the PREDICT tool [3] based on clinicopathologic variables is extensively recommended for estimating the survival of women with early-stage breast cancer following their surgical resection [4, 5]. An abundance of models is also available for cardiovascular risk prediction, including the Framingham, SCORE, and QRISK models [6]. When applied and communicated properly, (risk) predictions from models allow patients and clinicians to weigh the pros and cons of available options (e.g., adjuvant therapies for breast cancer, or preventive actions related to behavior or medication for cardiovascular disease) and can thereby support shared decision-making [7].

**Fig. 1:**
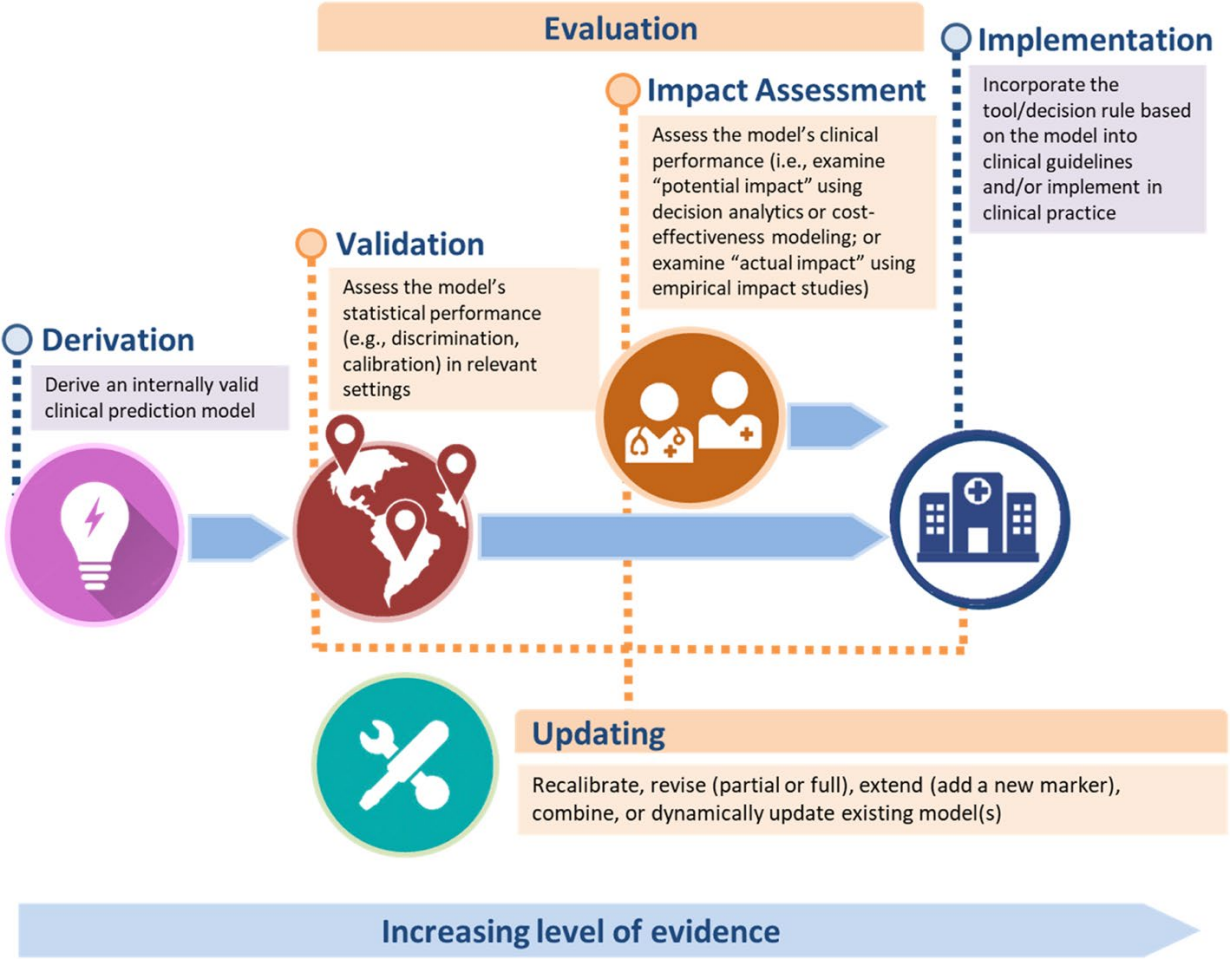
Framework from derivation to implementation of clinical prediction models. The focus of this systematic review is on model evaluation (validation, impact assessment) and updating

It is widely acknowledged [1, 2, 6, 8–10] that several steps must be taken to ensure that a sufficient level of evidence exists before adopting a prediction model in clinical practice (Fig. 1). Assessment of validity and impact generally constitute model “evaluation,” where the appropriateness and clinical consequences, respectively, of a model are examined [2, 8]. For instance, we may be interested in the suitability of PREDICT, a model derived from a predominantly white patient population, for multi-ethnic Asian patients [11]. We may also want to study how cardiovascular risk models influence clinical decision-making and patient outcomes, if at all [12]. After derivation, a model may be updated or re-specified, for instance, to incorporate new information from novel markers [13]. For example, PREDICT had been updated to incorporate a tumor proliferation marker (Ki-67) and several additional receptors (HER2 and progesterone) [14–16].

The illustrative examples mentioned so far are exceptions to the rule, as most models do not go beyond the initial development stage [6, 9, 17–20]. Reviews often find methodological shortcomings in validation studies [21–24] despite the publication of many methodological articles and reporting guidelines, foremost the Transparent Reporting of a multivariable prediction model for Individual Prognosis Or Diagnosis (TRIPOD) statement [25]. A topical example is the hundreds of models for SARS-CoV-2 (COVID-19), most of which are deemed useless due to inappropriate derivation and evaluation (i.e., calibration is ignored) [26]. Some less than ideal measures, such as the overall Net Reclassification Index (NRI), also remain popular in the medical literature despite being subject to severe statistical critique in the last decade [27–29]. On the same note, empirical evidence for the impact or usefulness of prediction models remains scarce [17, 30], perhaps due to the persisting ambiguity relating to the definition of the terms and confusion regarding applicable methods [31, 32]. Further clarification of terminologies and methods for model evaluation may benefit applied researchers.

Another widely recognized issue in prediction research is the incessant de novo derivation of models instead of refinement of existing ones [6, 18, 19]. Re-derivation (when acceptable models are already available) wastes research resources and potentially useful prior information. Moreover, unless settings completely differ, re-derivation may yield models with a similar set of core predictors for the same outcome. This then results in redundant models competing to address the same clinical problem. For breast cancer prognostication, around 60 models currently exist, of which the majority have the same set of predictors [18]. Likewise, a similar set of predictors can be found in over 300 models predicting cardiovascular disease risk [6], in over 40 models predicting prognosis for esophageal or gastric cancer [19], and so on. There is a clear need to stimulate research beyond model derivation, in particular towards studies that can be used to optimize existing prediction models for new settings [6, 18, 33]. A comprehensive summary of methods for model updating, including for newer types (e.g., meta-model [34] and dynamic updating [35]), is currently lacking.

We therefore aim to provide an overview of methodological guidance for the post-derivation stages of clinical prediction models. Specifically, we focus on methods for examining an existing model’s validity in specific settings, impact or usefulness in medical care, and model updating. Where appropriate, we outline consensus on definitions to support the methodological discussion, and we highlight gaps that require further research.

## Methods

### Literature search

We performed a systematic literature search in MEDLINE, Embase, Web of science core collection, Epistemonikos, Guidelines International Network, National Institute for Health and Care Excellence, Scottish Intercollegiate Guidelines Network, the STRengthening Analytical Thinking for Observational Studies initiative, and Google scholar in consultation with an experienced information specialist (W.S.) and a topic expert (E.W.S.). Snowballing was also conducted through references of shortlisted articles. Additional details (e.g., search terms, PRISMA workflow, PRISMA checklist) are available in the supplementary file (Additional file 1).

### Selection procedure and data synthesis

Articles were included if they 1) provided methodological “guidance” (i.e., standards, guidelines, frameworks, strategies, or recommendations) in model validation, impact assessment, or model updating; 2) were written in English; and 3) were published between January 2000 and January 2022. Abstracts, protocols, correspondence, supporting literature, and commentaries were excluded, as well as papers that discussed only one statistical technique or provided guidance not generalizable outside of a specific disease area. Initial selection based on title and abstract were conducted independently by two researchers (M.A.E.B. and E.G.E.), and any discrepancies were resolved through consensus meetings. Descriptive data (i.e., author(s), title, journal, case-study domain, codes/script availability, methodological topic(s) discussed) were extracted, and thematic analysis was used for summarization. Full text assessment and data extraction were performed by one researcher (M.A.E.B.). The results were reviewed by three researchers (E.W.S., M.K.S., E.G.E.) separately.

### Ethics statement

Ethics approval was not required for this review.

## Results

The search identified a total of 16,207 unique records (Additional file 1). After initial screening based on title and abstract, this number was reduced to 131 records. Snowballing from reference citations of shortlisted articles and expert consultation added an additional 51 records. After full text-assessment, 70 articles were included (Additional file 2). Approximately half of these (*n* = 34) were published in technical (statistical, bioinformatics, or methods research) journals. Many included at least one case-study (*n* = 43), often in the cardiovascular (*n* = 18) or cancer (*n* = 13) domain. Few articles included codes or scripts (*n* = 8), or mentioned that these were available upon request (n = 4). In the following sections, we present a summary of the retrieved methodological guidance. A glossary of common terms used throughout this review is available in Table 1.

**Table 1:** Definitions of some common terms used in this review

**Clinical prediction models** - sometimes referred to as clinical prediction rules, prediction algorithms, or risk scoring tools, are evidence-based tools that can aid in personalized medical decision making **Evaluation** - assessment of model validity (statistical performance) and impact (clinical performance) **Updating** - adjustment or re-specification of a model, e.g., to improve its performance in new data or incorporate new markers **Overfitting** - when model predictions are not valid for new subjects due to parameter uncertainty (i.e., uncertainty in predictor effects) or model uncertainty (e.g., selection of predictors may be biased) in the derivation data **Shrinkage** - a correction factor that can be applied to a model to address overfitting **Case-mix** - distribution of outcome and subject characteristics **Calibration drift** - miscalibration over time due, e.g., to changes in case-mix or clinical practice **Decision analysis** - a method used to assess clinical usefulness that takes into consideration different decisions or actions based on a clinical threshold or range of plausible thresholds **Individual participant data (IPD)** - raw, not aggregated or summarized, data	

### Validation

#### What is validation?

**Fig. 2:**
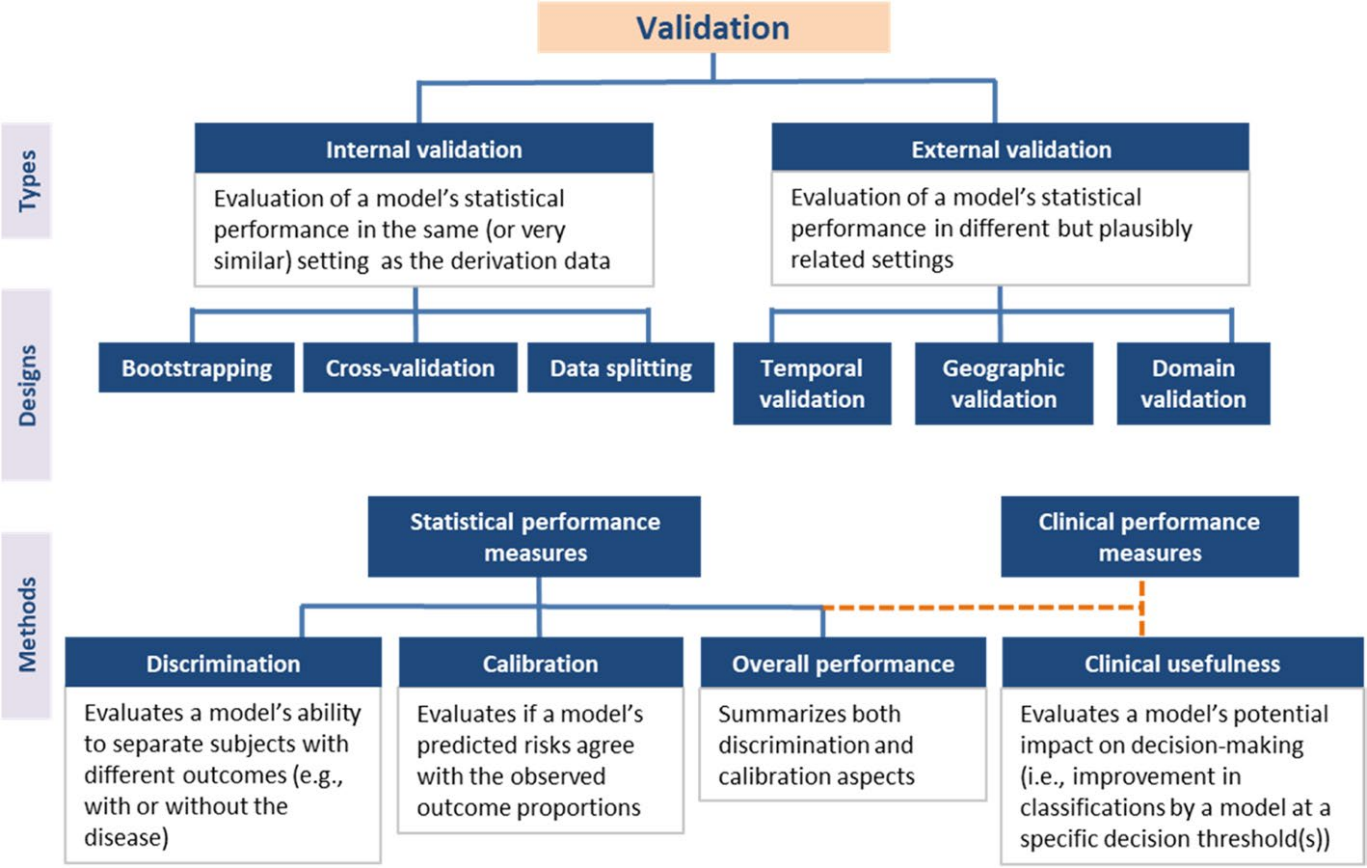
A summary of methodological guidance for model validation. Internal validation is the minimum requirement for clinical prediction models. External validation is recommended to evaluate model generalizability in different but plausibly related settings. Designs for validation studies differ in strength (e.g., temporal validation is a weak form of validation, and data splitting is discouraged). Examination of two validation aspects (discrimination and calibration) is recommended for assessing statistical performance irrespective of the type of validation. Clinical usefulness is a common area between validation and impact assessment, and its examination is advised for assessing the clinical performance of models intended to be used for medical decision-making

“Validation” is the process of assessing model performance in specific settings [13, 17, 25, 36–67]. Two types of validation are distinguished in the literature (Fig. 2).

Internal validation evaluates the reproducibility of model performance in subjects from the same data source or underlying population as the derivation data [17, 25, 36–38, 41–43, 48–52, 54–56, 60–62, 67]. Frequently recommended approaches for this type of validation are resampling techniques such as bootstrapping [17, 25, 36–39, 41, 42, 48–52, 55, 56, 58, 60, 61, 64, 67] or cross-validation [13, 17, 25, 37–39, 42, 45, 48, 49, 52, 55, 56, 60, 61, 64, 67]. These enable researchers to assess the extent of overfitting (Table 1) in model performance and adjust for it if necessary, for instance by applying a shrinkage correction factor [13, 25, 37, 48]. Data splitting or split sampling, where a dataset is divided into training (derivation) and test (validation) subsets, is not recommended as it constrains the sample size at both model derivation and validation, leading to imprecise estimates of predictive performance [36, 37, 42, 45, 51, 52, 56–58, 60, 61, 64]. Internal validation techniques based on resampling do not have this constraint regarding sample size [25, 38, 51, 56, 60, 61, 64, 67].

External validation evaluates the generalizability or transportability of model performance to subjects from different but plausibly related settings [17, 25, 36–39, 41–43, 46, 48, 50–52, 54–58, 60–67]. Common approaches for external validation are “temporal validation” (i.e., validation in subjects from more recent time periods) [17, 25, 36, 37, 41–44, 50, 52, 54–56, 60–62, 64, 67], “geographic-” or “spatial validation” (i.e., validation in different locations) [25, 36, 37, 41, 43, 50, 52, 54–56, 60, 61, 67], and “domain validation” (i.e., validation in different clinical domains or settings, e.g., validating a model derived in secondary care in a primary care setting) [41, 50, 55, 61, 67]. Temporal validation is sometimes referred to as a weak form of external validation as there is only a difference in calendar time between the derivation and validation datasets [42, 61, 64]. It can be defined as a form of data splitting using time period as a selection criterion [37]. Domain validation is regarded as the strongest form of validation as it allows assessment of model performance in very different settings [41, 50, 52, 56].

#### When should validation be performed?

At the minimum, a derived model must be internally validated [17, 25, 41, 43, 48, 50–52, 60, 61]. Internal validity is a prerequisite for external validity, and external validity is a prerequisite for implementation in a new setting [17, 36, 37, 41, 48, 50–52, 54, 58, 60, 61, 64–66]. There is no consensus regarding how many cumulative validations are necessary to claim that a model has been sufficiently validated. Different settings can involve different contexts (e.g., practices, measurements) and case-mixes, which may affect model generalizability [37, 41, 42, 44, 54, 55, 64]. A model found to perform well in a primary (general) care setting, for example, needs additional validation in a secondary (more specialized) care setting, and vice versa [41, 44, 54]. Another relevant consideration is sample size [36, 37, 39, 41, 54, 60, 65, 66]. A rule of thumb requiring at least 100 events and 100 non-events (or even 200 events and 200 non-events) has been suggested to have sufficient (80%) power to detect if the predictive accuracy is different from a pre-specified null hypothesis value (e.g., calibration intercept of 0 and calibration slope of 1) [41, 57, 59–61, 64, 65, 67]. More sophisticated methods that additionally consider assumptions regarding the validation data (e.g., expected distribution of events and censoring) are now available [63, 65, 66]. Rather than power calculation, these newer methods focus on precise estimation of performance measures [65, 66].

#### How can validity be assessed?

External validation is ideally conducted by independent researchers [17, 25, 36–38, 42–45, 52, 54, 58, 60–65, 67]. For an impression of model generalizability, it is recommended to compare the contexts of the derivation and validation data [36, 37, 39, 44, 54, 55, 57, 61, 64]. Some differences may exist (e.g., different practices or data collection methods, such as radiology or pathology grading, pre-analytical work-up for laboratory measurements) that warrant external validation [36, 56, 61]. Comparing case-mixes (including outcome distribution) is useful as a first step [36, 55, 58, 64]. Case-mix heterogeneity (e.g., difference in prognosis or disease severity) may support that the validation is a stronger test for transportability rather than reproducibility [13, 37, 41, 42, 44, 48–50, 54–57, 59–61, 64].

Several performance aspects can be examined in a validation study, with various measures proposed for each (see Additional file 3 for a more complete list):

1. Discrimination: A model discriminates well if it can separate subjects with different outcomes (e.g., subjects with and without events) [40, 47, 48, 51, 52, 54, 57, 61, 64–67]. The ideal model predicts risks close to 100% in subjects with the outcome and close to 0% in subjects without the outcome [38, 59]. Discrimination can be graphically assessed from boxplots, histograms, or density plots of predicted risks across outcome values, with better discriminating models showing less overlap [38, 48]. Calibration plots also graphically manifests discrimination from the spread in predictions [48, 52]. Discrimination can be quantified using the concordance (C-) statistic, which is identical to the area under the receiver operating characteristic curve (AUROC) in the logistic setting [38, 40, 45, 47, 48, 51, 52, 56, 57, 59–62, 64–67]. In survival settings, drawing an ROC curve is problematic as censored subjects have unknown outcomes; however, the C-statistic can be calculated with consideration to censoring [51, 52]. An alternative is the D-statistic, which can be interpreted as the log hazard ratio comparing two predicted risk groups defined by splitting the prognostic index (or linear predictor) at the median value [45, 46, 54, 57, 66]. Discrimination slope is another simple measure that calculates the absolute difference in average predicted risks for subjects with and without the outcome [48]. Classification measures such as sensitivity and specificity require the definition of a risk threshold that turns a model into a decision rule [38, 60, 61, 68], as such, they are discussed in the next section (see “Impact assessment”). Of note, statistical measures for discrimination may perform poorly when the case-mix is too homogeneous, for instance, when patients have less extreme predictions or narrower range of values for strong predictors [38, 45, 46, 48, 49, 51, 52, 56, 57]. It is advised to consider case-mix variation when interpreting or summarizing discrimination measures [46].

2. Calibration: Calibration refers to the agreement between predicted risks and observed outcomes [38, 40, 48, 51, 59–61, 64–67]. Complete model specification (including information about baseline risk) is needed to assess calibration [13, 46, 54, 64, 66]. To examine calibration graphically, calibration plots with or without some form of smoothing (e.g., LOESS, splines) are often recommended [38, 45, 47–52, 55–57, 59–65, 67]. In linear regression, the calibration plot is identical to a scatter plot [48, 63]. In logistic and survival settings, calibration is slightly more complex. The predicted risks are derived by applying the model to the data, and the observed “risks” are proportions of observed outcomes or events grouped according to quantiles of predicted risk [38, 42, 52, 57]. The agreement between the predicted risks and observed outcomes can be quantified using calibration-in-the-large and calibration slope [13, 38, 42, 48, 52, 54–57, 59, 60, 64–66]. Calibration-in-the-large compares the average predicted risk and observed outcome proportion, and indicates whether the predictions are systematically too high or too low [38, 59, 64–66]. It can be assessed using the calibration intercept [13, 38, 48, 52, 54–56, 59, 60, 65] or the O/E ratio [40, 57, 65]. Calibration slope indicates the extremeness of predicted risks and can be obtained by regression of the linear predictor to the data [13, 38, 42, 48, 52, 54–57, 59, 60, 63–66]. A calibration slope < 1 indicates that the predicted risks are too extreme (i.e., low risks are estimated to be too low, and high risks are estimated to be too high), while a slope > 1 indicates the opposite [52, 63, 65]. Overfitted models often yield a slope < 1, reflecting lack of generalizability and a need for correction in the validation setting [38, 48, 55, 63–65]. A slope < 1 may also indicate inconsistency of predictor effects (regression coefficients) between the derivation and validation settings [55]. It is recommended to report at least the calibration-in-the-large and calibration slope, with visualization to a calibration plot [52, 59, 65]. Use of the Hosmer-Lemeshow goodness-of-fit test is widely discouraged due to its limited power and poor interpretability [38, 42, 45, 47, 48, 51, 52, 59–62, 64].

3. Overall performance (or global fit): Overall performance measures encapsulate both discrimination and calibration aspects [37, 38, 45, 48, 53, 61]. The most commonly recommended measure is explained variation (R^2^), with variants such as Nagelkerke’s R^2^ for generalized linear models [45, 48, 61, 63]. For survival models, there is no consensus on which pseudo-R^2^ measure to use despite various proposals (e.g., Royston and Sauerbrei’s R^2^ based on the D-statistic, Schemper and colleagues’ explained variation) [45, 54]. Another measure is the Brier score, which is similar to Pearson’s R^2^ statistic when scaled and can be extended for survival outcomes using a weight function [37, 38, 40, 48, 54, 61, 69–71]. Information-theoretic approach such as the Akaike Information Criterion (AIC) and Bayesian Information Criterion (BIC) can also be used to estimate the trade-off between model fit and parsimony [61].

#### When is a model “good enough”?

It is recommended to examine at least two statistical performance aspects (discrimination and calibration) when assessing model validity in specific settings [13, 17, 25, 36–38, 41–45, 48–55, 57, 60–62, 64, 67]. If a model is to be used for risk stratification and limited resources are available such that high-risk patients need to be targeted, the focus should be on good discrimination [40, 48, 54, 64]. Poor discrimination is argued to be worse than poor calibration as the latter can be improved by model updating (e.g., recalibration) [13, 54]. Regarding what an adequate performance is, many authors agree that this depends on the context of application [13, 17, 36–38, 42, 48, 52–54, 64–66]. No minimum thresholds exist for discrimination (e.g., higher C-statistic is always better) and calibration (i.e., the closer the predictions are to the diagonal (45°) line, the better) [36, 38, 40, 42, 45, 47–52, 54–57, 59–62, 64, 65, 67, 69, 70, 72–75]. The minimum threshold for useful models can only be defined by examining decision-analytic measures (e.g., using the Net Benefit).

### Impact assessment

#### What is impact assessment?

Models with good statistical performance do not automatically have positive impact or usefulness in medical care [44, 50, 58, 60, 61, 68, 69, 76–78]. A model with good discrimination and calibration can be deemed clinically useless if it offers no new insight to existing clinical policy or has a range of predictions largely outside of clinically relevant decision thresholds [38, 48]. There may be various external factors (e.g., varying clinical interpretations, adherence, or acceptability) that may cause the actual performance of a model, or a decision rule developed based on a model, to differ from expectations [41, 61, 68, 76, 78]. The consequences of using a model in practice – what is referred to as “clinical impact” – need to be examined [17, 37, 38, 41, 43, 44, 48–50, 52, 53, 58, 60, 61, 64, 67–69, 72, 73, 76–80]. Common outcomes of interest for impact assessment are changes in clinicians’ decision-making or behaviors [17, 38, 41, 43, 44, 48–50, 58, 61, 68, 69, 72, 73, 76–80], patient outcomes (e.g., morbidity, mortality, quality of life, adverse effects) [17, 41, 43, 44, 49, 50, 52, 53, 58, 60, 61, 68, 69, 72, 76–80], and healthcare costs [17, 41, 44, 50, 52, 53, 58, 60, 61, 68, 69, 72, 73, 76, 77, 79].

#### How can impact be assessed?

**Fig. 3:**
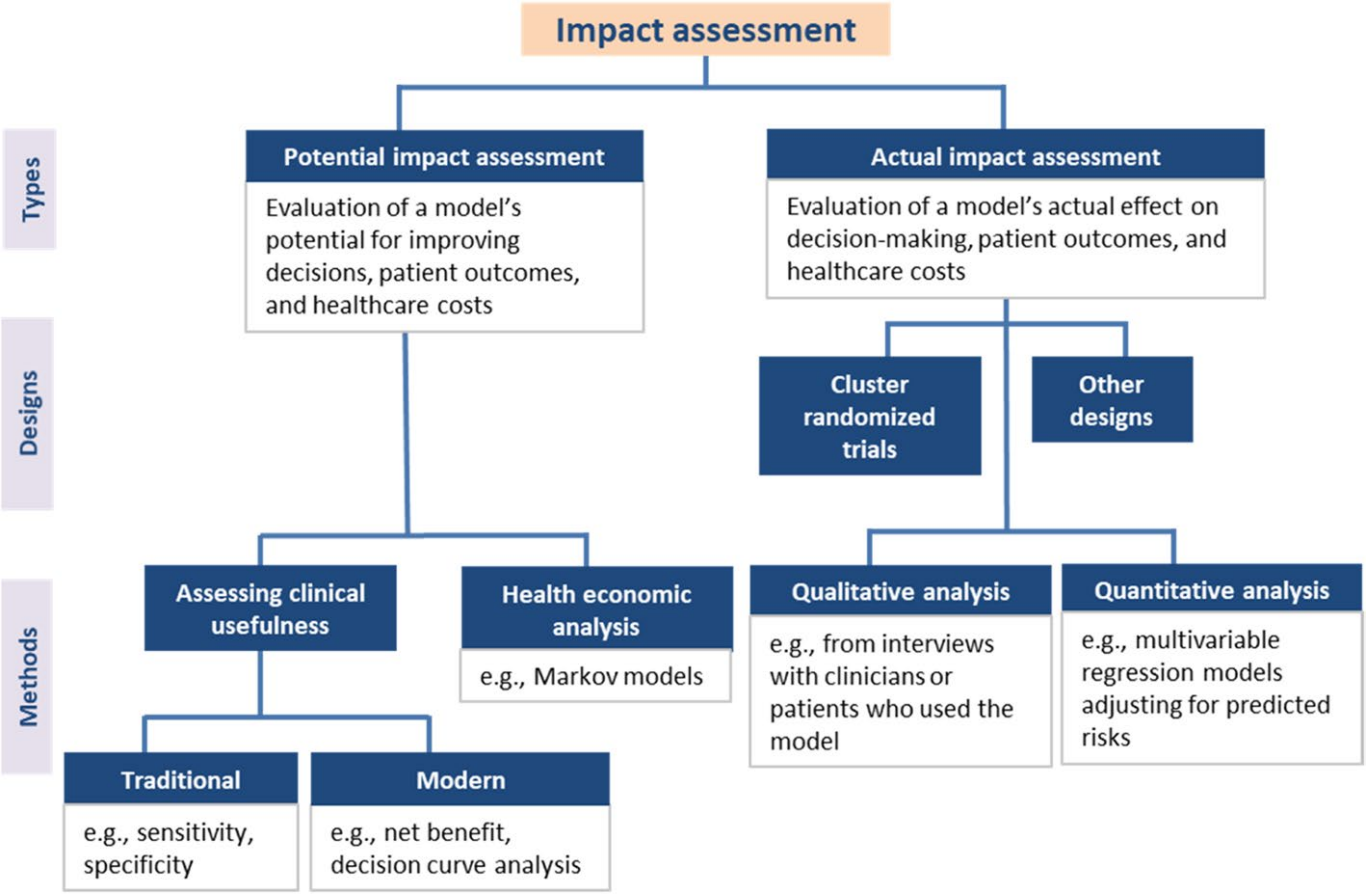
A summary of methodological guidance for the assessment of a model’s impact. Potential impact can be examined through clinical performance measures (e.g., Net Benefit, Decision Curve Analysis) or health economic analysis (e.g., decision-analytic models). Assessing actual impact requires comparative empirical studies, such as cluster randomized trials or other designs (e.g., stepped-wedge trials, before-after studies)

Impact assessment requires the definition of a decision threshold(s) at which subjects are classified into specific risk categories [38, 48, 52, 65, 66, 72]. At this threshold(s), a balance between the likelihood of benefit and harm exists [38, 52, 69]. Two types of impact assessment can be performed: potential and actual impact assessment (Fig. 3). The former evaluates theoretical changes on health outcomes through examination of clinical performance measures or decision-analytic models, while the latter evaluates actual effect using empirical studies [17, 44, 49, 50, 61, 69, 78].

1. Potential impact:
  i. Assessment of clinical usefulness – A simple approach for assessing a model’s potential impact is by examination of an aspect called “clinical usefulness” or “clinical utility” [17, 38, 44, 48–50, 52, 60, 61, 65, 67–69, 73, 77, 78]. While discrimination and calibration measures evaluate statistical performance, clinical usefulness measures (Additional file 3) evaluate clinical or practical performance [38, 60]. Given a specific threshold, clinical usefulness can be examined using classification measures such as sensitivity and specificity [48, 61]. These measures along with similar variants (e.g., positive and negative predictive values) and simplistic summaries (e.g., accuracy) depend on outcome prevalence and are not recommended to be reported in isolation [38, 53, 60, 61, 68, 69]. More recent papers recommend using decision-analytic measures, such as the Net Benefit [47, 48, 52, 60, 61, 65–67, 69, 73, 78], which take the clinical consequences (i.e., relative weight of benefits and harms) into account. In particular, the Net Benefit quantifies clinical benefit by penalizing true positive classifications with the harms of false-positive classifications [60, 65, 69]. It is recognized that a clinically accepted threshold may not exist and that the weights of benefits and harms may vary across patients or clinicians (e.g., some patients are willing to take a higher risk for a possible benefit) [48, 69]. Hence, a sensitivity analysis of the Net Benefit over an array of thresholds (i.e., Decision Curve Analysis (DCA)) is also recommended, with visualization to a decision curve [47, 48, 52, 60, 69]. A variant of a decision curve is the relative utility curve, where net benefit is scaled by comparing it to the case of perfect prediction [61, 65, 69, 73].
  ii. Health economic analysis – A more comprehensive assessment of potential impact is through decision-analytic models (e.g., Markov models, decision trees or simulations assessing cost-effectiveness) [17, 44, 50, 61, 73, 78, 81, 82]. Development of such models is recommended as an intermediate step when conducting a formal impact study is not yet feasible due to time or cost constraints [44, 61, 78]. If the projected results are poor (e.g., high costs per quality-adjusted life year), some argue that empirical impact studies may not be warranted [17, 44, 50, 78]. Decision-analytic models ideally undergo further validation [81]. Decision-analytic measures such as the Net Benefit can be re-expressed to incorporate specific cost (e.g., test or treatment costs) and effectiveness (e.g., life expectancy) parameters, placing them one step closer to a full health economic analysis [73, 82].
2. Actual impact: The actual impact of using a model or, alternatively, a decision rule based on a model, can be assessed in a cluster randomized trial [17, 41, 44, 50, 53, 60, 61, 64, 67–69, 76–79]. Other designs, such as stepped-wedge trial, before-after study, or cross-sectional study, although less preferred, can also be used [17, 41, 44, 50, 61, 67, 68, 78]. Analyses from impact studies compare the outcomes of a group where a model is used versus a control group that uses no model or care-as-usual [17, 41, 44, 50, 61, 67, 68, 76–78]. Evaluation can be performed in an assistive or directive manner [44, 50, 58, 61, 68, 76, 78]. An assistive approach leaves more room to combine model predictions with clinicians’ judgements or interpretations, while a directive approach suggests a specific decision or action based on a model’s risk classification (as is the case when a model is translated into a decision rule) [44, 50, 58, 61, 68, 76, 78]. Findings from impact studies (e.g., practical barriers to implementation) can be used to improve the prediction model or rule, develop clinical guidelines, or inform the development of new models [17, 41, 58, 61, 68, 76].

#### When can impact be assessed?

Some clinical contexts may not require decisions and reporting of impact (e.g., a model intends only to inform patients of a likely outcome, such as the chance of a successful pregnancy) [47, 60]. Many agree that impact assessment is important for models intended to be used for decision-making (e.g., a model that guides decisions regarding administration of chemotherapy or of an invasive or expensive test) [17, 41, 47, 48, 50, 52, 65, 68, 76, 78]. Impact assessment (when warranted) is recommended after external validation and ideally prior to model implementation [17, 38, 41, 44, 48–50, 53, 60, 61, 68, 69, 76–79].

### Updating

#### What is model updating and when is it useful?

Many authors recognize that new models are too often derived [34, 41, 42, 44, 50, 54, 55, 57, 59–61, 83–86]. If models have already been proposed for a prediction problem, then deriving a model from scratch is a waste of information from previous modeling studies [13, 34, 41, 44, 50, 60, 61, 83, 84, 87–90]. Furthermore, it is noted that relatively small datasets are sometimes used in re-derivation, resulting in overfitted new models that may not perform well elsewhere [34, 41, 44, 59, 61, 83, 86, 87, 91]. If a reasonable model is already available that produces well-discriminating (although perhaps slightly miscalibrated) estimates, the consensus is to build upon such a model and check if some adjustments (“updating”) will improve its fit or performance in new data [13, 17, 25, 34, 41, 44, 50, 54, 55, 57, 59–61, 64, 71, 83–94]. Model updating can also be a way to incorporate a novel marker into a model (model extension) [13, 17, 41, 44, 50, 55, 60, 61, 71, 87–91, 94] or to address a model’s miscalibration over time (calibration drift) due, for instance, to changes in patient demographics or clinical practice [17, 41, 42, 59, 83, 87, 89, 90, 93–98]. The updated model ideally undergoes further evaluation [13, 17, 34, 41, 42, 50, 54, 61, 71, 83–93, 95].

#### How can models be updated?

**Fig. 4:**
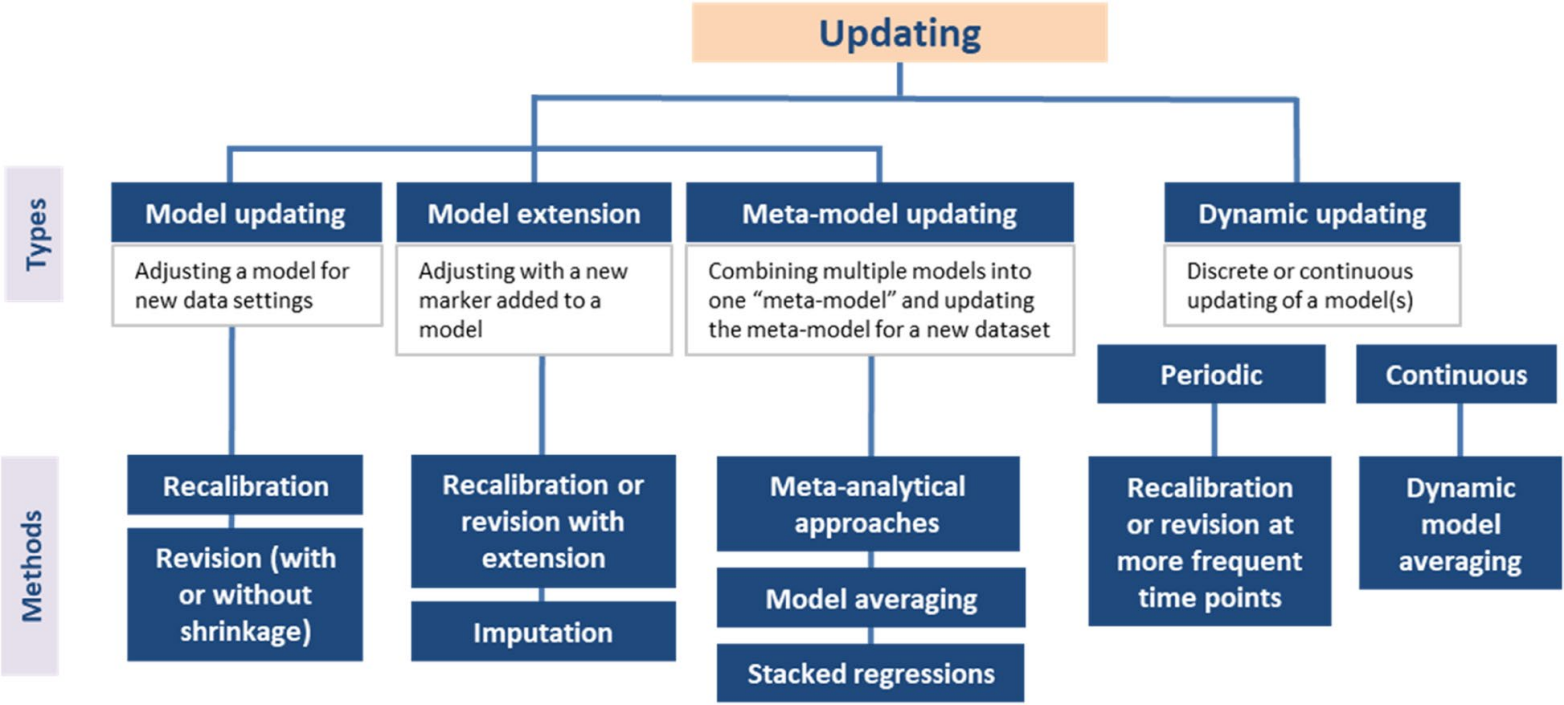
A summary of methodological guidance for model updating. Simple updating (e.g., recalibration) is often sufficient when the differences between the derivation and new data are minimal. When the differences are more substantial, more extensive updating (e.g., partial to full revision) may be appropriate. Model extension allows the incorporation of new markers in a model. Multiple models may be combined, for instance, using meta-analytical approaches, to develop a meta-model that can be further updated for a new dataset. Updating can also be done periodically or continuously, resulting in dynamically updated models

The literature distinguishes four types of model updating for regression-based models (Fig. 4). Updating methods for more computationally-intensive models (e.g., deep neural networks) were not identified.

1. Model updating – Updating can start with predictions from the original model [13, 50, 86, 87]. Recommended methods range from recalibration (i.e., using the linear predictor of the original model and subsequently optimizing its fit in new data) to model revision (i.e., re-estimation of some or all of the coefficients (predictor effects) from the original model) [13, 17, 25, 34, 41, 42, 44, 50, 54, 55, 57, 59–61, 71, 83, 86, 87, 89–91, 93, 94, 96]. Recalibration addresses at least the difference in baseline risks by re-estimating the model intercept or baseline hazard (“recalibration-in-the-large”) [61, 86, 89]. A further step is to recalibrate the linear predictor of the original model (termed “logistic recalibration” in the logistic setting) [50, 61, 86, 94]. Closed-testing procedures have recently been proposed to aid researchers in selecting the optimal updating approach in logistic [86] and multinomial logistic settings [90]. In a closed-test procedure, the fit of the updated models are compared with each other using likelihood ratio tests [86]. Preference is then given to the most conservative approach that yields a significant improvement in model fit [86]. Recalibration is often sufficient when the differences between the derivation and new datasets are minimal (i.e., calibration is the main issue in new data) [13, 44, 64, 71, 83, 86, 89–91, 93, 96]. More extensive methods, such as model revision, are appropriate when the differences are more substantial (e.g., predictor effects are heterogeneous leading to inconsistent predictions in the new dataset) [55, 86, 90, 96]. As model revision requires re-estimation of more parameters than recalibration, it requires a larger sample size [59, 90, 96] and potentially the application of shrinkage methods to compensate for overfitting [71, 96]. Bayesian approaches are applicable regardless of the sample size [89, 93].

2. Model extension – An interest may be in adding a new marker to an existing model with specific predictors [13, 17, 41, 44, 50, 55, 60, 61, 71, 87–89, 91, 94]. Such model extension is a more extensive type of model updating [13, 71, 90, 91, 96]. Some recommended methods are recalibration or revision with extension and Bayesian updating [13, 55, 71, 88, 91, 94]. As with simpler updating, overfitting can be mitigated using shrinkage methods or by limiting the number of estimated parameters [71, 91]. If the individual participant data (IPD) is available for the model’s original derivation data, it has been suggested to impute the marker in a combined (derivation and marker) dataset, and thereafter derive an updated model [91]. This approach assumes that the derivation and marker datasets originate from the same underlying population [91].

The incremental value of a marker can be studied by comparing the performance of the extended and original (non-extended) models [69, 74]. Specifically, improvements in discrimination (e.g., increase in C-statistic) and overall performance measures (e.g., decrease in Brier score, increase in R^2^) can be examined [17, 45, 48, 51, 52, 69, 70, 72–75, 79]. Likelihood ratio test and its approximations (e.g., Wald test) can be used to test the improvement in model fit due to the addition of a new marker [75, 79]. The additional potential clinical impact or usefulness of a marker can be assessed using decision-analytic measures such as the Net Benefit [69, 99]. Reclassification measures (Additional file 3), specifically the overall Net Reclassification Index (NRI) and Integrated Discrimination Index (IDI), are not recommended as these measures apply weights based on outcome prevalence alone rather than on the clinical consequences of decisions [45, 48, 51, 52, 60, 69, 72, 73]. The actual impact of incorporating a new marker can be assessed through impact studies [17, 52, 69, 73, 79]. Calibration is less relevant if a marker is being assessed in the dataset where the model was originally derived and for which the model is already well-calibrated [69]. On the other hand, if the original model is poorly calibrated for a new (marker) dataset, the apparent performance increment attributable to the marker can be over or under-estimated [60, 75]. One paper argues that refitting of the original model to the marker dataset is necessary prior to assessing a marker’s incremental value [75].

3. Meta-model updating – Multiple models may have already been published to predict the same outcome. Combining these models into one could potentially yield a more generalizable “meta-model” [13, 34, 87]. This meta-model can then be updated for a new dataset [34, 57, 84]. If the available models have similar specifications (i.e., same predictors), meta-analytic (e.g., univariate or multivariate with random effects) or Bayesian approaches can be used to produce a meta-model [84, 87]. When published models have different specifications (i.e., different predictors), methods such as model averaging and stacked regressions have been proposed [34]. Although theoretically appealing, meta-model updating is still a relatively new idea with limited methodological discussion [34, 57, 84, 85, 87, 92].

4. Dynamic updating – “Static” models are derived from a single time period in a single dataset [97]. There is increasing interest in “dynamically updated models” that may more efficiently mitigate calibration drift [87, 89, 93, 95, 97, 98]. Note that such dynamic updating is different from dynamic modeling that focuses on including time-dependent covariates [13, 95]. For periodically updated models, updating can be performed using conventional methods such as recalibration and revision [89, 93, 95, 97, 98]. For continuously updated models, methods such as Bayesian dynamic modeling or dynamic model averaging (generalization to multiple models) have been suggested [87, 95, 98]. The application of a Kalman filter with a weight-decay function has also been proposed to incorporate changes over time [95]. Here, the significance of a “forgetting factor” is highlighted, with historic patients given less weight compared to newer patients [95]. Similar to meta-model updating, there is currently limited literature for dynamic updating [13, 87, 89, 93, 95, 97]. Framework is also lacking for further validation of dynamic models [95].

## Discussion

Clinical prediction models are evidence-based tools that can aid in personalized medical decision-making. As with any scientific tool, their applicability and usefulness are ideally evaluated prior to their clinical adoption. Suboptimal performance may be improved by model adjustment or re-specification, for instance, to incorporate additional information from a specific setting or to include new markers. We aimed to provide a summary of contemporary methodological guidance for the evaluation (validation and impact assessment) and updating of clinical prediction models. To our knowledge, this is the first comprehensive review of guidance for these post-derivation stages.

We found many methodological guidance papers for model evaluation. The consensus for validation (assessment of statistical performance) is that it requires at least the examination of discrimination and calibration measures (Additional file 3). For discrimination, concordance statistics (e.g., Harrell’s or Uno’s C-statistic) are considered sufficient for indicating how well a model can distinguish different outcomes [48, 49, 54, 55]. We did not find recommendations in favor of alternatives that are common in the machine learning literature, such as the precision-recall curve (PRC) [100]. For calibration, it is recommended to report at least the mean calibration (i.e., calibration-in-the-large, measured using the O/E ratio or calibration intercept) and extremeness of predicted risks (i.e., calibration slope) [65]. Visualization of calibration measures to a calibration plot is also regarded as useful for checking (mis)calibration over the entire range of predictions [38, 65].

Assessment of impact or usefulness (clinical performance) is deemed necessary for models intended to be used for medical decision-making. Recommendations have shifted from simplistic classification measures (e.g., accuracy, overall NRI) to decision-analytic measures that additionally incorporate clinical consequences (e.g., Net Benefit, DCA) [101, 102]. More recent developments to decision-analytic measures include proposals to incorporate actual costs and effectiveness parameters to further bridge decision analytics and full health economic analysis [73, 82]. The weighted NRI (Additional file 3), which applies the same decision-analytic weighting as the Net Benefit [69, 99], has also been proposed as an alternative to the overall NRI. Other decision-analytic measures exist, albeit with less popularity in the methodological literature. Examples include decrease in weighed false classifications [38], loss functions (compares loss associated with classification errors [40]), test trade-off (gives the minimum number of new marker tests needed for a true positive to produce an increase in the net benefit [103–105]), and relative utility (compares net benefit to the case of perfect prediction [61, 69, 73]).

Complete model specification is highlighted as crucial for the evaluation, particularly, the assessment of calibration of prediction models [13, 46, 54]. We found that miscalibration in new data can be resolved using simple updating methods, such as recalibration-in-the-large (i.e., re-estimation of the model intercept or baseline hazard) and/or adjustment of the slope [106]. More extensive updating (i.e., partial to full model revision) is recommended when predictor effects differ substantially between the derivation and new datasets [55, 86, 90]. We identified closed-testing procedures [86, 90] that can help researchers in selecting an appropriate updating method in the logistic and multinomial logistic settings.

Some gaps remain in the methodological literature. For instance, guidance is lacking for calculating the minimum necessary sample size for external validation of machine learning-based models [63, 65, 66]. Guidance for updating is limited to regression-based models only, where extensions are lacking for, for example, the validation of dynamic prediction models. We did not identify caveats for model updating when the clinical setting is not ideal (e.g., very effective treatments are used for high-risk patients defined by the prediction model). We also did not identify methods for retiring or replacing predictors that may have lost their clinical significance over time. Further research and additional guidance are necessary in these areas.

We acknowledge several limitations in our review. First, papers published before 2000 were not included unless cited by the shortlisted articles. While we cannot rule out that some methodological articles may have been missed, we find it unlikely that major recommendations were not incorporated in the selected papers. Some bias, however, may be present due to the choice of search terms and our definition of what constitutes “guidance.” Next, our review is limited by what we retrieved from the selected literature. For instance, some performance measures were not discussed by the retrieved articles and hence were not included in this review (e.g., variants to R^2^ such as Cox-Snell’s [107] or McFadden’s [108], variants of the concordance statistic [109–112], test trade-off [103–105]). Beyond the scope of our review, commentaries and more specific statistical papers also exist, which further elucidate on the interrelations of different performance measures, for example, the effect of miscalibration on the Net Benefit [113] and other classification measures (e.g., NRI, IDI) [114, 115]. Finally, we noticed a lack of methods particular for machine learning-based models [116–118]. While a few papers argue that the same general methodological considerations apply [59, 64], we are aware that at least some terminologies differ (e.g., predictors are called features, bootstrap aggregation is called bagging). Some recommendations are also different (e.g., split-sampling design is recommended for internal validation of complex modeling procedures [67], and discrimination is assessed by precision-recall curves (PRC), which have some similarity to receiver operating characteristic (ROC) curves). It may therefore be informative to conduct a more targeted review that will enable a critical comparison of definitions and methodological standards between traditional regression and machine learning-based models. We recognize that efforts are underway to develop reporting guidelines for machine learning-based models [119], which may help standardize concepts and methods.

## Conclusion

The post-derivation stages of clinical prediction models are important for optimizing model performance in new settings that may be contextually different from or beyond the scope of the initial model development. Substantial methodological guidance is available for model evaluation (validation and impact assessment) and updating. For model evaluation, we found that performance measures based on decision analysis provide additional practical insight beyond statistical performance (discrimination and calibration) measures. For model updating, we identified various methods including recalibration, revision, and extension. Additional guidance is necessary for machine learning-based models and relatively new types of updating, such as meta-model and dynamic updating. Our summary can be used as a starting point for researchers who want to perform post-derivation research or critique published studies of similar nature.

## Supplementary Information

**Additional file 1.** Full methodology. Description of methods and related information.

**Additional file 2: Table S1.** Overview of selected articles included in the review.

**Additional file 3: Table S2.** Summary of performance measures from the selected methodological literature.

## Data Availability

All data generated and analyzed during this review are included in the manuscript and its additional files.

## Abbreviations

AUROC: Area Under the Receiver Operating Characteristic curve
DCA: Decision Curve Analysis
IPD: Individual Participant Data
LOESS: LOcally Estimated Scatterplot Smoothing
NRI: Net Reclassification Index
PRC: Precision-Recall Curve
PRISMA: Preferred Reporting Items for Systematic Reviews and Meta-Analyses
