# Supplementary material for: Methodological guidance for the evaluation and updating of clinical prediction models: a systematic review
Source: BMC Med Res Methodol. 2022 Dec 12;22:316. doi: 10.1186/s12874-022-01801-8 (PMC9742671; doi:10.1186/s12874-022-01801-8)
Supplement: Supplementary file 1 — Additional file 1. Full methodology. Description of methods and related information. [file 12874_2022_1801_MOESM1_ESM.docx]

**DESCRIPTION OF METHODS AND RELATED INFORMATION**

**CONTENTS**

[**METHODS** 2](#_Toc103701142)

[**DATABASES** 4](#_Toc103701143)

[**SEARCH TERMS** 5](#_Toc103701144)

[**PRISMA WORKFLOW** 11](#_Toc103701145)

[**PRISMA WORKFLOW (EXTENSION)** 12](#_Toc103701146)

[**PRISMA 2020 CHECKLIST** 13](#_Toc103701147)

# **METHODS**

**Registration:**

This systematic review was registered in PROSPERO with ID [CRD42020210650](https://www.crd.york.ac.uk/prospero/display_record.php?ID=CRD42020210650).

**Search strategy:**

The literature search was performed in MEDLINE, Embase, Web of science core collection, Epistemonikos, Guidelines International Network, National Institute for Health and Care Excellence, Scottish Intercollegiate Guidelines Network, and the STRengthening Analytical Thinking for Observational Studies initiative, in consultation with an experienced information specialist (W.S.). This was supplemented by a search in Google scholar, references from the shortlisted articles, and expert consultation (E.W.S.).

The initial search was limited to articles published from January 2000 to September 2020. An updated search was performed to include publications until January 2022. Search strategies were tailored to each database and included terms synonymous or relating to (a) “guidance,” (b) “prediction model,” (c) “model evaluation, validation, or impact assessment,” and (d) “model updating, and extension with a new marker”.

**Selection procedure:**

Selection of papers was performed in two phases. In the first phase, titles and abstracts were screened for eligibility by two independent reviewers (M.A.B. and E.G.E.). Initial screening was blinded and was performed using the Rayyan application [1]. Discrepancies were resolved through consensus meetings between the two reviewers, and doubt regarding final selection was resolved through discussion with two researchers (E.W.S., M.K.S.). In the second phase, additional databases and references cited by the shortlisted articles and recommendations by a topic expert (E.W.S.) were searched. A final eligibility check was conducted, and the same method for resolving discrepancies was applied.

Articles were included if they 1) provided methodological “guidance” (i.e., standards, guidelines, frameworks, strategies, or recommendations) in model validation, impact assessment, or model updating; 2) were written in English; and 3) were published between January 2000 and January 2022. Abstracts, protocols, correspondence, supporting literature, and commentaries were excluded, as well as papers that discussed only one statistical technique or provided guidance not generalizable outside of a specific disease area.

**Data extraction and analysis:**

Descriptive data (i.e., author(s), title, journal, case-study domain, codes/script availability, methodological topic(s) discussed) were extracted. Thematic analysis was used to summarize the selected guidance papers. Data extraction was performed by one researcher (M.A.B.). The results were reviewed by three researchers (E.W.S., M.K.S., E.G.E.) separately.

**Quality assessment:**

To our knowledge, there is currently no grading criteria applicable to methodological guidance. While we could have developed our own quality or bias assessment criteria, for instance by modifying those intended for clinical practice guidelines [2, 3], their application would have distracted from the main focus of the review (synthesis rather than grading). With these considerations, the quality of the selected guidance was not formally assessed, but a strict selection procedure was applied.

# **DATABASES**

*Date: 01-Jan-2000 up to 10-Jan-2022*

1. MEDLINE
2. Embase
3. Web of Science Core Collection
4. Epistemonikos
5. Guidelines International Network (GIN)
6. National Institute for Health and Care Excellence (NICE)
7. Scottish Intercollegiate Guidelines Network (SIGN)
8. STRengthening Analytical Thinking for Observational Studies (STRATOS) initiative
9. Google scholar (first 20 pages/200 hits)

*Date: Not applicable*

1. References from the shortlisted articles
2. Recommendations from a topic expert (E.W.S.)

# **SEARCH TERMS**

1. **MEDLINE**

| \| [*# ▲*](http://ovidsp.dc2.ovid.com/ovid-a/ovidweb.cgi?&S=AOBGFPDKJDEBEKLMIPAKDHEHOHGHAA00&Sort+Sets=descending) \| Searches \| Results \| \| --- \| --- \| --- \| \| 1 \| exp guideline/ or ("recommendation*" or "protocol*" or "consensus" or "statement*" or "position paper*" or "guideline*" or "guidance*" or "framework*" or "strategy" or "strategies" or "methodological standard*").ti. \| 483901 \| \| 2 \| *decision support techniques/ or *Models, Theoretical/ or *Risk Assessment/ or ((decision adj (support* or aid or analys* or tree*)) or "theoretical model*" or "risk assess*" or ((predict* or risk or prognostic or diagnostic or "decision making") adj (model* or rule* or outcome* or score* or scoring or tool*))).ti. \| 165946 \| \| 3 \| evaluation study/ or exp evaluation studies as topic/ or validation study/ or (validit* or validat* or evaluat* or assess* or performance or impact or implication or discrimination or calibration or utility or "cost effect*" or "cost analys*" or "cost benefit*" or reporting).ti,ab,kf. \| 8932117 \| \| 4 \| ("updat*" or "extension*" or "adjustment*" or "addition*" or ((new or added) adj (variable* or predictor* or marker* or factor* of covariate*))).ti,ab,kf. \| 3472812 \| \| 5 \| 3 or 4 \| 10968357 \| \| 6 \| 1 and 2 and 5 \| 4327 \| \| 7 \| limit 6 to yr="2000 -Current" \| 4037 \| |
| --- | --- | --- | --- | --- | --- | --- | --- | --- | --- | --- | --- | --- | --- | --- | --- | --- | --- | --- | --- | --- | --- | --- | --- | --- |

1. **Embase**

| \| [*# ▼*](https://amc-literatuur.amc.nl/f5-w-687474703a2f2f6f76696473702e6463312e6f7669642e636f6d$$/ovid-a/ovidweb.cgi?&S=CNCCFPPJNMACEKAGKPAKOGGJNKOPAA00&Sort+Sets=ascending) \| Searches \| Results \| \| --- \| --- \| --- \| \| 7 \| limit 6 to yr="2000 -Current" \| 8071 \| \| 6 \| 1 and 2 and 5 \| 8508 \| \| 5 \| 3 or 4 \| 14051930 \| \| 4 \| ("updat*" or "extension*" or "adjustment*" or "addition*" or ((new or added) adj (variable* or predictor* or marker* or factor* of covariate*))).ti,ab,kw. \| 4582979 \| \| 3 \| exp evaluation study/ or validation study/ or (validit* or validat* or evaluat* or assess* or performance or impact or implication or discrimination or calibration or utility or "cost effect*" or "cost analys*" or "cost benefit*" or reporting).ti,ab,kw. \| 11449527 \| \| 2 \| *decision support system/ or *theoretical model/ or *risk assessment/ or ((decision adj (support* or aid or analys* or tree*)) or "theoretical model*" or "risk assess*" or ((predict* or risk or prognostic or diagnostic or "decision making") adj (model* or rule* or outcome* or score* or scoring or tool*))).ti. \| 177824 \| \| 1 \| exp practice guideline/ or ("recommendation*" or "protocol*" or "consensus" or "statement*" or "position paper*" or "guideline*" or "guidance*" or "framework*" or "strategy" or "strategies" or "methodological standard*").ti. \| 1059598 \| |
| --- | --- | --- | --- | --- | --- | --- | --- | --- | --- | --- | --- | --- | --- | --- | --- | --- | --- | --- | --- | --- | --- | --- | --- | --- |

1. **Web of Science Core Collection**

| Set | Results | Save History / Create AlertOpen Saved History |
| --- | --- | --- |
| # 6 | 6,465 | #5 AND #2 AND #1  Indexes=SCI-EXPANDED, SSCI, A&HCI, ESCI Timespan=2000-2022 |
| # 5 | 21,758,495 | #4 OR #3  Indexes=SCI-EXPANDED, SSCI, A&HCI, ESCI Timespan=2000-2022 |
| # 4 | 6,041,870 | TOPIC: (("updat*" or "extension*" or "adjustment*" or "addition*" or ((new or added) adj (variable* or predictor* or marker* or factor* of covariate*) )))  Indexes=SCI-EXPANDED, SSCI, A&HCI, ESCI Timespan=2000-2022 |
| # 3 | 18,776,993 | TOPIC: ((validit* or validat* or evaluat* or assess* or performance or impact or implication or discrimination or calibration or utility or "cost effect*" or "cost analys*" or "cost benefit*" or reporting) )  Indexes=SCI-EXPANDED, SSCI, A&HCI, ESCI Timespan=2000-2022 |
| # 2 | 345,505 | TITLE: (((decision adj (support* or aid or analys* or tree*) ) or "theoretical model*" or "risk assess*" or ((predict* or risk or prognostic or diagnostic or "decision making") adj (model* or rule* or outcome* or score* or scoring or tool*) )))  Indexes=SCI-EXPANDED, SSCI, A&HCI, ESCI Timespan=2000-2022 |
| # 1 | 902,090 | TITLE: ("recommendation*" OR "protocol*" OR "consensus" OR "statement*" OR "position paper*" OR "guideline*" OR "guidance*" OR "framework*" OR "strategy" OR "strategies" OR "methodological standard*")  Indexes=SCI-EXPANDED, SSCI, A&HCI, ESCI Timespan=2000-2022 |

1. **Epistemonikos**

| #1 | Title/Abstract | ("recommendation*" OR "protocol*" OR "consensus" OR "statement*" OR "position paper*" OR "guideline*" OR "guidance*" OR "framework*" OR "strategy" OR "strategies" OR "methodological standard*") |
| --- | --- | --- |
| #2 | Title/Abstract | ("theoretical model*" OR "risk assess*" OR "predicting model*" OR "predicting rule*" OR "predicting outcome*" OR "predicting score*" OR "predicting scoring" OR "predicting tool*" OR "predictive model*" OR "predictive rule*" OR "predictive outcome*" OR "predictive score*" OR "predictive scoring" OR "predictive tool*" OR "prediction model*" OR "prediction rule*" OR "prediction outcome*" OR "prediction score*" OR "prediction scoring" OR "prediction tool*" OR "risk model*" OR "risk rule*" OR "risk outcome*" OR "risk score*" OR "risk scoring" OR "risk tool*" OR "prognostic model*" OR "prognostic rule*" OR "prognostic outcome*" OR "prognostic score*" OR "prognostic scoring" OR "prognostic tool*" OR "diagnostic model*" OR "diagnostic rule*" OR "diagnostic outcome*" OR "diagnostic score*" OR "diagnostic scoring" OR "diagnostic tool*" OR "decision making model*" OR "decision making rule*" OR "decision making outcome*" OR "decision making score*" OR "decision making scoring" OR "decision making tool*") |
| #3 | #1 AND #2 | 1642 |
| #4 | Filters: min year=2000, max year=2022 | 1,608 |

[Filters: min_year=2000, max_year=2022]

1. **Guidelines International Network (GIN)**

**Initial search:**

We found **17** results out of **3955** entries for your search.

validit* or validat* or evaluat* or assess* or performance or impact or implication or discrimination or calibration or utility or "cost effect*" or "cost analys*" or "cost benefit*" or reporting or "updat*" or "extension*" or "adjustment*" or "addition*" or "new variable*" or "added variable*" or "new predictor*" or "added predictor*" or "new marker*" or "added marker*" or "new factor*" or "added factor*" or "new covariate*" or "added covariate*"

Filters:

- Language > English
- Publication > Systematic review / evidence report / guideline clearing report / guideline methodology / implementation tool

**Updated search:**

Since the initial search, the library had been changed and could no longer be searched with truncated keywords and/or Boolean operators. Therefore, all guidelines published in 2020 and 2021 (295) were downloaded and searched using the same keywords. No new publications for inclusion were found.

1. **National Institute for Health and Care Excellence (NICE)**

**Initial search:**

("theoretical model" or "theoretical models" or "predicting model" or "predicting models" or "predictive model" or "predictive models" or "prediction model" or "prediction models" or "risk model" or "risk models" or "prognostic model" or "prognostic models" or "diagnostic model" or "diagnostic models" or "decision making model" or "decision making models" or "decision making tool" or "decision making tools") and (validity or validating or validation or evaluation or evaluate or evaluating or evaluations or update or updating or updates or extension or extensions or adjustment or adjustments or addition or additions or additional)

**Updated search:**

Similar to GIN, the system was updated to a newer version that did not allow for an advanced search using specific search fields and/or Boolean operators. Instead, a manual selection (i.e., a simple search using single words plus date filter) of the publications released since the former search date was made. The same keywords as the original search were used. This retrieved no results.

1. **Scottish Intercollegiate Guidelines Network (SIGN)**

<https://www.sign.ac.uk/what-we-do/publications/>

1. **STRengthening Analytical Thinking for Observational Studies (STRATOS) initiative**

<https://www.stratos-initiative.org/publications>

1. **Google scholar (first 20 pages/200 hits)**

**Initial search:**

(guideline*)("theoretical|predict*|risk|prognostic|diagnostic|decision making)(model*|tool*|rule*|outcome*|scoring|score*)(validit*|validat*|evaluat*|assess*|performance|impact|implication|discrimination|calibration|utility|"cost effect*"|"cost analys*"|"cost benefit*"|updat*|extens*|adjust*|addition*)

**Updated search:**

Since the initial search, Google’s use of wildcards and maximum length of search string had changed. This caused issues when using the original search terms. The search terms were therefore adapted.

(guideline|guidelines|guidance|framework|standard|recommendation)(theoretical|predict|risk|prognostic|diagnostic|decision making)(model|tool|rule|outcome|scoring|score)(validity|validation|evaluation|assessment|performance|impact|implication|discrimination|calibration|utility|"cost effectiveness|updating|adjustment|extension|marker)

# **PRISMA WORKFLOW**

**Included**

**Eligibility**

**Screening**

**Identification**

Records after

duplicates removed

(n=14,513)

Records excluded (n=14,416)

Remaining papers after

title and abstract screening (n=97)

Full-text articles excluded (n=52):

- Commentary (n=4)
- Duplicate/same article in a different journal (n=40)
- Not in English (n=2)
- Out of scope (n=6)

Records identified through database searching

(n=18,542)

Additional records identified from snowballing (reference citations) + expert consultation

(n=39)

Full text articles included

(n=58)

Full-text articles excluded (n=26):

- Already included (n=10)
- Commentary (n=2)
- Limited to one statistical technique (n=8)
- Not article (n=1)
- Out of scope (5)

Flow diagram of the initial search (from January 2000 to September 2020) according to the Preferred Reporting Items for Systematic Reviews and Meta-Analyses (PRISMA) statement [4].

# **PRISMA WORKFLOW (EXTENSION)**

**Included**

**Eligibility**

**Screening**

**Identification**

Records after previous search results removed

(n=4,325)

Records excluded (n=4,291)

Remaining papers after

title and abstract screening (n=34)

Full-text articles excluded (n=29):

- Correspondence (n=2)
- Commentary (n=1)
- Duplicate (n=8)
- Limited to one statistical technique (n=1)
- Not article (n=1)
- Protocol (n=2)
- Out of scope (n=14)

Records after duplicates removed

(n=16,207)

Additional records identified from snowballing (reference citations) + expert consultation

(n=12)

Full text articles included

(n=12)

Full-text articles excluded (n=5):

- Correspondence (n=1)
- Commentary (n=2)
- Out of scope (n=2)

Records identified through database searching

(n=20,418)

Flow diagram of the updated search (from September 2020 until January 2022) according to the Preferred Reporting Items for Systematic Reviews and Meta-Analyses (PRISMA) statement [4].

# **PRISMA 2020 CHECKLIST**

| **Section and Topic** | **Item #** | **Checklist item** | **Location where item is reported *** |
| --- | --- | --- | --- |
| **TITLE** | | |  |
| Title | 1 | Identify the report as a systematic review. | Title |
| **ABSTRACT** | | |  |
| Abstract | 2 | See the PRISMA 2020 for Abstracts checklist. | Abstract |
| **INTRODUCTION** | | |  |
| Rationale | 3 | Describe the rationale for the review in the context of existing knowledge. | Background, paragraphs 3-4 |
| Objectives | 4 | Provide an explicit statement of the objective(s) or question(s) the review addresses. | Background, paragraph 5 |
| **METHODS** | | |  |
| Eligibility criteria | 5 | Specify the inclusion and exclusion criteria for the review and how studies were grouped for the syntheses. | Methods, paragraph 2; Additional file 1; Additional file 2 |
| Information sources | 6 | Specify all databases, registers, websites, organisations, reference lists and other sources searched or consulted to identify studies. Specify the date when each source was last searched or consulted. | Methods, paragraph 1; Additional file 1 |
| Search strategy | 7 | Present the full search strategies for all databases, registers and websites, including any filters and limits used. | Additional file 1 |
| Selection process | 8 | Specify the methods used to decide whether a study met the inclusion criteria of the review, including how many reviewers screened each record and each report retrieved, whether they worked independently, and if applicable, details of automation tools used in the process. | Methods, paragraph 2; Additional file 1 |
| Data collection process | 9 | Specify the methods used to collect data from reports, including how many reviewers collected data from each report, whether they worked independently, any processes for obtaining or confirming data from study investigators, and if applicable, details of automation tools used in the process. | Methods, paragraph 2; Additional file 1 |
| Data items | 10a | List and define all outcomes for which data were sought. Specify whether all results that were compatible with each outcome domain in each study were sought (e.g. for all measures, time points, analyses), and if not, the methods used to decide which results to collect. | Methods, paragraph 2; Additional file 1 |
|  | 10b | List and define all other variables for which data were sought (e.g. participant and intervention characteristics, funding sources). Describe any assumptions made about any missing or unclear information. | NA |
| Study risk of bias assessment | 11 | Specify the methods used to assess risk of bias in the included studies, including details of the tool(s) used, how many reviewers assessed each study and whether they worked independently, and if applicable, details of automation tools used in the process. | NA |
| Effect measures | 12 | Specify for each outcome the effect measure(s) (e.g. risk ratio, mean difference) used in the synthesis or presentation of results. | NA |
| Synthesis methods | 13a | Describe the processes used to decide which studies were eligible for each synthesis (e.g. tabulating the study intervention characteristics and comparing against the planned groups for each synthesis (item #5)). | Methods, paragraph 2; Additional file 1 |
|  | 13b | Describe any methods required to prepare the data for presentation or synthesis, such as handling of missing summary statistics, or data conversions. | Methods, paragraph 2; Additional file 1 |
|  | 13c | Describe any methods used to tabulate or visually display results of individual studies and syntheses. | Methods, paragraph 2; Additional file 1 |
|  | 13d | Describe any methods used to synthesize results and provide a rationale for the choice(s). If meta-analysis was performed, describe the model(s), method(s) to identify the presence and extent of statistical heterogeneity, and software package(s) used. | Methods, paragraph 2; Additional file 1 |
|  | 13e | Describe any methods used to explore possible causes of heterogeneity among study results (e.g. subgroup analysis, meta-regression). | NA |
|  | 13f | Describe any sensitivity analyses conducted to assess robustness of the synthesized results. | NA |
| Reporting bias assessment | 14 | Describe any methods used to assess risk of bias due to missing results in a synthesis (arising from reporting biases). | NA |
| Certainty assessment | 15 | Describe any methods used to assess certainty (or confidence) in the body of evidence for an outcome. | NA |
| **RESULTS** | | |  |
| Study selection | 16a | Describe the results of the search and selection process, from the number of records identified in the search to the number of studies included in the review, ideally using a flow diagram. | Results, paragraph 1; Additional file 1 |
|  | 16b | Cite studies that might appear to meet the inclusion criteria, but which were excluded, and explain why they were excluded. | Additional file 1 |
| Study characteristics | 17 | Cite each included study and present its characteristics. | Additional file 2 |
| Risk of bias in studies | 18 | Present assessments of risk of bias for each included study. | NA |
| Results of individual studies | 19 | For all outcomes, present, for each study: (a) summary statistics for each group (where appropriate) and (b) an effect estimate and its precision (e.g. confidence/credible interval), ideally using structured tables or plots. | NA |
| Results of syntheses | 20a | For each synthesis, briefly summarise the characteristics and risk of bias among contributing studies. | Results, paragraph 1; Additional file 2 |
|  | 20b | Present results of all statistical syntheses conducted. If meta-analysis was done, present for each the summary estimate and its precision (e.g. confidence/credible interval) and measures of statistical heterogeneity. If comparing groups, describe the direction of the effect. | NA |
|  | 20c | Present results of all investigations of possible causes of heterogeneity among study results. | NA |
|  | 20d | Present results of all sensitivity analyses conducted to assess the robustness of the synthesized results. | NA |
| Reporting biases | 21 | Present assessments of risk of bias due to missing results (arising from reporting biases) for each synthesis assessed. | NA |
| Certainty of evidence | 22 | Present assessments of certainty (or confidence) in the body of evidence for each outcome assessed. | NA |
| **DISCUSSION** | | |  |
| Discussion | 23a | Provide a general interpretation of the results in the context of other evidence. | Discussion, paragraphs 1-6 |
|  | 23b | Discuss any limitations of the evidence included in the review. | Discussion, paragraph 5 |
|  | 23c | Discuss any limitations of the review processes used. | Discussion, paragraph 6 |
|  | 23d | Discuss implications of the results for practice, policy, and future research. | Conclusion |
| **OTHER INFORMATION** | | |  |
| Registration and protocol | 24a | Provide registration information for the review, including register name and registration number, or state that the review was not registered. | Additional file 1 |
|  | 24b | Indicate where the review protocol can be accessed, or state that a protocol was not prepared. | Additional file 1 |
|  | 24c | Describe and explain any amendments to information provided at registration or in the protocol. | NA |
| Support | 25 | Describe sources of financial or non-financial support for the review, and the role of the funders or sponsors in the review. | Manuscript (“Funding”) |
| Competing interests | 26 | Declare any competing interests of review authors. | Manuscript (“Competing interests”) |
| Availability of data, code and other materials | 27 | Report which of the following are publicly available and where they can be found: template data collection forms; data extracted from included studies; data used for all analyses; analytic code; any other materials used in the review. | Manuscript (“Availability of data and materials”) |
| * NA = Not applicable | | | |

1. Ouzzani, M., et al., *Rayyan-a web and mobile app for systematic reviews.* Syst Rev, 2016. **5**(1): p. 210.

2. Brouwers, M.C., et al., *AGREE II: advancing guideline development, reporting and evaluation in health care.* J Clin Epidemiol, 2010. **63**(12): p. 1308-11.

3. Medicine, I.o., *Clinical Practice Guidelines We Can Trust*, ed. R. Graham, et al. 2011, Washington, DC: The National Academies Press. 290.

4. Liberati, A., et al., *The PRISMA statement for reporting systematic reviews and meta-analyses of studies that evaluate healthcare interventions: explanation and elaboration.* BMJ, 2009. **339**: p. b2700.
