# Supplementary material for: Methodological guidance for the evaluation and updating of clinical prediction models: a systematic review
Source: BMC Med Res Methodol. 2022 Dec 12;22:316. doi: 10.1186/s12874-022-01801-8 (PMC9742671; doi:10.1186/s12874-022-01801-8)
Supplement: Supplementary file 2 — Additional file 2: Table S1. Overview of selected articles included in the review. [file 12874_2022_1801_MOESM2_ESM.docx]

**OVERVIEW OF SELECTED ARTICLES INCLUDED IN THE QUALITATIVE SYNTHESIS**

|  |  |  |  |  | **Topic(s) discussed** | | |
| --- | --- | --- | --- | --- | --- | --- | --- |
| **Author(s)/Year** | **Title** | **Journal** | **Case-study domain** | **Codes/script availability *** | **Model validation** | **Model impact assessment** | **Model updating †** |
| Altman (2009) | Prognostic models: a methodological framework and review of models for breast cancer | Cancer Investigation | Cancer - Breast | No or NA | **✓** |  |  |
| Altman and Royston (2000) | What do we mean by validating a prognostic model? | Statistics in Medicine | Severe head injury; Asthma; Acute myocardial infarction; Falls in elderly patients | No or NA | **✓** | **✓** |  |
| Altman et al. (2009) | Prognosis and prognostic research: validating a prognostic model | BMJ - Research Methods & Reporting | Mortality after cardiac surgery; Mortality after colorectal surgery; Mortality for failure of ventilation in patients with COPD; Complications of acute cough in children | No or NA | **✓** |  | **✓** |
| Ankerst et al. (2012) | Updating risk prediction tools: a case study in prostate cancer | Biometrical Journal | Cancer - Prostate | Yes, R |  |  | **✓** |
| Archer et al. (2021) | Minimum sample size for external validation of a clinical prediction model with a continuous outcome | Statistics in Medicine | No specific case study; various examples mentioned | No or NA | **✓** |  |  |
| Austin et al. (2016) | Geographic and temporal validity of prediction models: different approaches were useful to examine model performance | Journal of Clinical Epidemiology | Congestive heart failure | No or NA | **✓** |  |  |
| Bleeker et al. (2003) | External validation is necessary in prediction research: A clinical example | Journal of Clinical Epidemiology | Serious bacterial infection in children with fever without source | No or NA | **✓** |  |  |
| Bossuyt et al. (2012) | Beyond diagnostic accuracy: the clinical utility of diagnostic tests | Clinical Chemistry | No specific case study; various examples mentioned | No or NA |  | **✓** |  |
| Boulesteix and Sauerbrei (2011) | Added predictive value of high-throughput molecular data to clinical data and its validation | Briefings in Bioinformatics | No specific case study; various examples mentioned | No or NA |  |  | **✓** |
| Collins et al. (2015) | Transparent reporting of a multivariable prediction model for Individual Prognosis or Diagnosis (TRIPOD): the TRIPOD statement | Journal of Clinical Epidemiology (and others) | No specific case study; various examples mentioned | No or NA | **✓** |  | **✓** |
| Cowley et al. (2019) | Methodological standards for the development and evaluation of clinical prediction rules: a review of the literature | Diagnostic and Prognostic Research | No specific case study; various examples mentioned | No or NA | **✓** | **✓** | **✓** |
| de Hond et al. (2022) | Guidelines and quality criteria for artificial intelligence-based prediction models in healthcare: a scoping review | npj Digital Medicine | None | No or NA | **✓** | **✓** | **✓** |
| Debray et al. (2012) | Aggregating published prediction models with individual participant data: a comparison of different approaches | Statistics in Medicine | Traumatic brain injury; Deep venous thrombosis | Available upon request |  |  | **✓** |
| Debray et al. (2013) | A framework for developing, implementing, and evaluating clinical prediction models in an individual participant data meta-analysis | Statistics in Medicine | Deep venous thrombosis | Available upon request |  |  | **✓** |
| Debray et al. (2014) | Meta-analysis and aggregation of multiple published prediction models | Statistics in Medicine | Deep venous thrombosis | No or NA |  |  | **✓** |
| Debray et al. (2015) | Individual participant data (IPD) meta-analyses of diagnostic and prognostic modeling studies: guidance on their use | Public Library of Science Medicine | No specific case study; various examples mentioned | No or NA |  |  | **✓** |
| Debray et al. (2015) | A new framework to enhance the interpretation of external validation studies of clinical prediction models | Journal of Clinical Epidemiology | Deep venous thrombosis | No or NA | **✓** |  | **✓** |
| Dekker et al. (2017) | Con: Most clinical risk scores are useless | Nephrology Dialysis Transplantation | No specific case study; various examples mentioned | No or NA | **✓** | **✓** |  |
| Dent et al. (2012) | Risk prediction models: a framework for assessment | Public Health Genomics | No specific case study; various examples mentioned | No or NA | **✓** | **✓** |  |
| Gail and Pfeiffer (2005) | On criteria for evaluating models of absolute risk | Biostatistics | Cancer - Breast | No or NA | **✓** | **✓** |  |
| Hemingway et al. (2009) | Ten steps towards improving prognosis research | BMJ - Research Methods & Reporting | No specific case study; various examples mentioned | No or NA |  | **✓** | **✓** |
| Hickey et al. (2013) | Dynamic Prediction Modeling Approaches for Cardiac Surgery | Circulation: Cardiovascular Quality and Outcomes | Mortality after cardiac surgery | No or NA |  |  | **✓** |
| Hlatky et al. (2009) | Criteria for evaluation of novel markers of cardiovascular risk: a scientific statement from the American Heart Association | Circulation | Cardiovascular disease | No or NA |  | **✓** | **✓** |
| Janssen et al. (2008) | Updating methods improved the performance of a clinical prediction model in new patients | Journal of Clinical Epidemiology | Severe postoperative pain | No or NA |  |  | **✓** |
| Janssens et al. (2011) | Strengthening the reporting of genetic risk prediction studies: The GRIPS statement | Annals of Internal Medicine | No specific case study; various examples mentioned | No or NA |  |  | **✓** |
| Jenkins et al. (2018) | Dynamic models to predict health outcomes: current status and methodological challenges | Diagnostic and Prognostic Research | No specific case study; various examples mentioned | No or NA |  |  | **✓** |
| Justice et al. (1999) ‡ | Assessing the generalizability of prognostic information | Annals of Internal Medicine | Cancer - Colon | No or NA | **✓** |  |  |
| Kappen et al. (2012) | Adaptation of Clinical Prediction Models for Application in Local Settings | Medical Decision Making | Postoperative nausea and vomiting | No or NA |  |  | **✓** |
| Kappen et al. (2018) | Evaluating the impact of prediction models: lessons learned, challenges, and recommendations | Diagnostic and Prognostic Research | Postoperative nausea and vomiting | No or NA |  | **✓** |  |
| Katki and Bebu (2021) | A simple framework to identify optimal cost-effective risk thresholds for a single screen: Comparison to Decision Curve Analysis | Journal of the Royal Statistical Society Series A (Statistics in Society) | Cancer - Breast and ovarian | No or NA |  | **✓** | **✓** |
| Kattan and Gerds (2020) | A Framework for the Evaluation of Statistical Prediction Models | Chest | No specific case study; various examples mentioned | No or NA | **✓** |  | **✓** |
| Kearns et al. (2013) | Good practice guidelines for the use of statistical regression models in economic evaluations | Pharmacoeconomics | No specific case study; various examples mentioned | No or NA |  | **✓** |  |
| McGeechan et al. (2008) | Assessing new biomarkers and predictive models for use in clinical practice: A clinician's guide | Archives of Internal Medicine | Coronary heart disease | No or NA |  | **✓** | **✓** |
| McShane et al. (2006) | REporting recommendations for tumor MARKer prognostic studies (REMARK) | Breast Cancer Research and Treatment | No specific case study; various examples mentioned | No or NA |  |  | **✓** |
| Mijderwijk et al. (2022) | Updating Clinical Prediction Models: An Illustrative Case Study | Acta Neurochirurgica | Postoperative anxiety | No or NA |  |  | **✓** |
| Moons et al. (2009) | Prognosis and prognostic research: application and impact of prognostic models in clinical practice | BMJ - Research Methods & Reporting | No specific case study; various examples mentioned | No or NA | **✓** | **✓** | **✓** |
| Moons et al. (2009) | Prognosis and prognostic research: what, why, and how? | BMJ - Research Methods & Reporting | No specific case study; various examples mentioned | No or NA | **✓** | **✓** |  |
| Moons et al. (2012) | Risk prediction models: I. Development, internal validation, and assessing the incremental value of a new (bio)marker | Heart | Cardiovascular disease | No or NA | **✓** |  | **✓** |
| Moons et al. (2012) | Risk prediction models: II. External validation, model updating, and impact assessment | Heart | Cardiovascular disease / Coronary heart disease | No or NA | **✓** | **✓** | **✓** |
| Nieboer et al. (2016) | Improving prediction models with new markers: a comparison of updating strategies | BMC Medical Research Methodology | Cancer - Prostate | Yes, R |  |  | **✓** |
| Pencina et al. (2008) | Evaluating the added predictive ability of a new marker: From area under the ROC curve to reclassiﬁcation and beyond | Statistics in Medicine | Coronary heart disease | No or NA |  |  | **✓** |
| Pencina et al. (2011) | Extensions of net reclassification improvement calculations to measure usefulness of new biomarkers | Statistics in Medicine | Coronary heart disease | No or NA |  |  | **✓** |
| Ramspek et al. (2021) | External validation of prognostic models: what, why, how, when and where? | Clinical Kidney Journal | No specific case study; various examples mentioned | No or NA | **✓** | **✓** | **✓** |
| Rapsomaniki et al. (2012) | A framework for quantifying net benefits of alternative prognostic models | Statistics in Medicine | Cardiovascular disease | No or NA |  | **✓** | **✓** |
| Reilly and Evans (2006) | Translating clinical research into clinical practice: impact of using prediction rules to make decisions | Annals of Internal Medicine | No specific case study; various examples mentioned | No or NA |  | **✓** |  |
| Riley et al. (2016) | External validation of clinical prediction models using big datasets from e-health records or IPD meta-analysis: opportunities and challenges | BMJ - Research Methods & Reporting | No specific case study; various examples mentioned | No or NA | **✓** |  | **✓** |
| Riley et al. (2021) | Minimum sample size for external validation of a clinical prediction model with a binary outcome | Statistics in Medicine | Heart failure | Yes, Stata | **✓** | **✓** |  |
| Riley et al. (2022) | Minimum sample size calculations for external validation of a clinical prediction model with a time-to-event outcome | Statistics in Medicine | Venous thromboembolism | Yes, Stata and R | **✓** | **✓** |  |
| Royston and Altman (2013) | External validation of a Cox prognostic model: principles and methods | BMC Medical Research Methodology | Cancer - Breast | No or NA | **✓** |  | **✓** |
| Schnellinger et al. (2021) | Comparison of dynamic updating strategies for clinical prediction models | Diagnostic and Prognostic Research | Lung transplant | Available upon request |  |  | **✓** |
| Siregar et al. (2016) | Improved Prediction by Dynamic Modeling - An Exploratory Study in the Adult Cardiac Surgery Database of the Netherlands Association for Cardio-Thoracic Surgery | Circulation: Cardiovascular Quality and Outcomes | Mortality after cardiac surgery | No or NA |  |  | **✓** |
| Siregar et al. (2019) | Methods for updating a risk prediction model for cardiac surgery: a statistical primer | Interactive CardioVascular and Thoracic Surgery | Mortality after cardiac surgery | No or NA |  |  | **✓** |
| Steyerberg and Vergouwe (2014) | Towards better clinical prediction models: seven steps for development and an ABCD for validation | European Heart Journal | Acute myocardial infarction | Yes, R (available in a linked website) | **✓** | **✓** | **✓** |
| Steyerberg et al. (2004) | Validation and updating of predictive logistic regression models: a study on sample size and shrinkage | Statistics in Medicine | Acute myocardial infarction | No or NA |  |  | **✓** |
| Steyerberg et al. (2010) | Assessing the performance of prediction models: a framework for some traditional and novel measures | Epidemiology | Cancer - Testicular | Yes, R | **✓** | **✓** | **✓** |
| Steyerberg et al. (2012) | Assessing the incremental value of diagnostic and prognostic markers: a review and illustration | European Journal of Clinical Investigation | Cancer - Testicular | Yes, R (available in a linked website) |  | **✓** | **✓** |
| Steyerberg et al. (2013) | Prognosis Research Strategy (PROGRESS) 3: Prognostic Model Research | Public Library of Science | No specific case study; various examples mentioned | No or NA | **✓** | **✓** | **✓** |
| Su et al. (2018) | A review of statistical updating methods for clinical prediction models | Statistical Methods in Medical Research | Mortality after cardiac surgery | Available upon request |  |  | **✓** |
| Toll et al. (2008) | Validation, updating and impact of clinical prediction rules: A review | Journal of Clinical Epidemiology | No specific case study; various examples mentioned | No or NA | **✓** | **✓** | **✓** |
| Van Calster et al. (2017) | Validation and updating of risk models based on multinomial logistic regression | Diagnostic and Prognostic Research | Pregnancies of unknown location | No or NA |  |  | **✓** |
| Van Calster et al. (2019) | Calibration: the Achilles heel of predictive analytics | BMC Medicine | No specific case study; various examples mentioned | No or NA | **✓** |  | **✓** |
| van Houwelingen (2000) | Validation, calibration, revision and combination of prognostic survival models | Statistics in Medicine | Cancer - Non-Hodgkin’s lymphoma | No or NA | **✓** |  | **✓** |
| Vergouwe et al. (2002) | Validity of prediction models: when is a model clinically useful? | Seminars in Urologic Oncology | Cancer - Testicular and prostate | No or NA | **✓** | **✓** |  |
| Vergouwe et al. (2017) | A closed testing procedure to select an appropriate method for updating prediction models | Statistics in Medicine | Cancer - Prostate; Traumatic brain injury; Children presenting with fever | Yes, R |  |  | **✓** |
| Vickers and Cronin (2010) | Everything you always wanted to know about evaluating prediction models (but were too afraid to ask) | Urology | No specific case study; various examples mentioned | No or NA | **✓** | **✓** |  |
| Vickers and Cronin (2010) | Traditional statistical methods for evaluating prediction models are uninformative as to clinical value: towards a decision analytic framework | Seminars in Oncology | No specific case study; various examples mentioned | No or NA | **✓** | **✓** |  |
| Wallace et al. (2011) | Framework for the impact analysis and implementation of Clinical Prediction Rules (CPRs) | BMC Medical Informatics and Decision Making | No specific case study; various examples mentioned | No or NA |  | **✓** |  |
| Wood and Greenland (2009) | Evaluating the prognostic value of new cardiovascular biomarkers | Disease Markers | No specific case study; various examples mentioned | No or NA | **✓** |  | **✓** |
| Wynants et al. (2017) | Key steps and common pitfalls in developing and validating risk models | BJOG – An International Journal of Obstetrics and Gynaecology | Cancer - Ovarian; Vaginal delivery after a previous caesarian section | No or NA | **✓** | **✓** | **✓** |
| Xanthakis et al. (2014) | Assessing the incremental predictive performance of novel biomarkers over standard predictors | Statistics in Medicine | Atrial fibrillation | No or NA |  |  | **✓** |
| * NA = Not applicable | | | | | | | |
| † Included articles that discussed the assessment of the incremental value of new markers on top of available predictors in a model, as part of model extension. | | | | | | | |
| ‡ One article, found through reference citations and published earlier than 2000, was included due to its relevance. | | | | | | | |
