# Supplementary material for: Methodological guidance for the evaluation and updating of clinical prediction models: a systematic review
Source: BMC Med Res Methodol. 2022 Dec 12;22:316. doi: 10.1186/s12874-022-01801-8 (PMC9742671; doi:10.1186/s12874-022-01801-8)
Supplement: Supplementary file 3 — Additional file 3: Table S2. Summary of performance measures from the selected methodological literature. [file 12874_2022_1801_MOESM3_ESM.docx]

**SUMMARY OF PERFORMANCE MEASURES**

**FROM THE SELECTED METHODOLOGICAL LITERATURE**

| **Aspects** | **Measures** | **Definitions** | **Interpretations** |
| --- | --- | --- | --- |
| **Overall performance** | Squared distance: Brier Score  [1-15] | Mean squared difference between observed outcomes and predictions | Ranges from 0 (perfect model) to 0.25 (worthless model with a 50% outcome prevalence, equivalent to a fair coin flip). This measure can be scaled to range from 0-1.  Note: For survival outcomes, a variant of the Brier score can be used (“Integrated Brier Score”). This applies a weighting function that considers censoring [4]. |
|  | Explained variation: R^2^  [2-4, 6, 16-18] | Proportion of outcome variation explained by the predictors in the model | Ranges from 0 to 1. The closer the value is to 1, the better the predictors in the model are able to explain the outcome variation.  Note: For survival outcomes, a variant called Nagelkerke’s R^2^ is sometimes used [4]. This pseudo-R^2^ measure is known to perform poorly in the presence of censoring and should be used with caution [19, 20]. Other alternatives have been proposed [19, 21], but there is no consensus on which to use. |
|  | Information criteria:  Akaike Information Criterion (AIC) and Bayesian Information Criterion (BIC)  [2, 22] | Trade-off between goodness of fit and model parsimony | AIC and BIC scores are useful in comparison with other AIC and BIC scores (different models in the same data). For both, a lower score indicates a better model fit. |
| **Discrimination** | Boxplot of predicted risks  [2, 4, 5] | Visualizes the spread in predictions | Predicted risks close to 100% in subjects with the outcome and predicted risks close to 0% in subjects without the outcome indicate good discrimination. A large overlap indicates poor discrimination.  Note: Instead of boxplot, histograms or density plots can be examined with the same interpretation [4]. |
|  | Concordance (C-) statistic or index, and Area Under ROC curve (AUROC)  [2-8, 12, 16, 22-40] | For binary (logistic) outcome, the C-statistic refers to the probability that a randomly drawn subject with the outcome had a higher risk than a subject without the outcome. For time-to-event (survival) outcome, this is generalized as the probability that the subject who had an event earlier had higher risk. | Values close to 1 indicate good discrimination, while values close to 0.5 indicate poor discrimination. A useless prediction model equivalent to a fair coin flip yields a C-statistic value of 0.5.  Note: In logistic settings, the C-statistic is identical to the AUROC. For survival outcomes, variants to Harrell’s C-statistic are available, such as Uno’s C-statistic, which does not ignore censored observations [12, 41]. A time-dependent AUROC can also be derived [42].  **Caveat**: A homogeneous case-mix may yield a low C-statistic [4, 5, 16, 27, 29-31, 36, 43]. ROC curves are deemed uninformative unless clinically relevant thresholds are indicated [4, 44, 45]. |
|  | D-statistic for survival outcomes  [3, 16, 29, 41, 43] | Measures prognostic separation | This can be interpreted as the log hazard ratio for two groups defined by splitting the prognostic index at the median value.  Similar to the C-statistic, higher values for the D-statistic indicate better discrimination.  **Caveat**: As with the C-statistic, a homogeneous case-mix may yield a lower D-statistic. |
|  | Discrimination slope  [2, 4] | Absolute difference in average predicted risks for subjects with and without the outcome | The larger the value (difference), the better the discrimination. |
| **Calibration** | Calibration plot  [2, 4, 5, 12, 16, 17, 22-32, 35-40, 46] | Graph of observed outcomes (y-axis) vs. predicted risks (x-axis) | If the observed outcomes and predicted risks are in agreement, the calibration plot shows a curve or a line close to the diagonal (45°) line.  Note: A calibration plot can be extended to a “validation plot” that demonstrates clinical usefulness through observations below or above a specific threshold value(s) [5, 30]. |
|  | Calibration intercept (α)  [3-5, 12, 24, 30, 32, 36, 38, 40, 46] | Estimate of systematically too high/low predicted risks (“calibration-in-the-large”) | Ideally, calibration intercept is 0. A calibration intercept below (above) 0 indicates that the model overestimates (underestimates) the outcome.  Note: In logistic settings, the calibration intercept (α) is calculated in a calibration model (e.g., log odds of an outcome = α + β*linear predictor) with the calibration slope (β) constrained to 1 [40]. For survival outcomes, calibration intercept is not directly calculated from a Cox model. |
|  | Calibration slope (β)  [3-5, 12, 17, 23, 24, 29, 30, 32, 36, 38, 40, 41, 46] | Estimate of extremeness of predicted risks | A calibration slope of 1 indicates good overall calibration, while below (above) 1 indicates too extreme (too restricted) risk estimation.  Note: Calibration slope (β) is calculated by regression of the linear predictor to the data (i.e., regression slope in a calibration plot) [40]. |
|  | O/E ratio  [8, 29, 40] | The ratio between observed and expected outcomes (related to the calibration-in-the-large) | The ideal ratio is 1, while below (above) 1 indicates that the model over- (under-) predicts risk.  Note: A variant is E/O ratio, where interpretation is reversed. The ratio can be reported for groups of predicted risks, from which the trend reflects the calibration slope [29]. |
|  | Hosmer-Lemeshow goodness-of-fit test  [2, 4, 5, 12, 16, 22, 23, 26, 27, 30, 32, 35, 37, 38] | Test for goodness-of-fit or deviance of grouped (e.g., deciles) observed outcomes and predicted risks | A high value for this test statistic is related to a small p-value and a general lack of fit.  **Caveat:** This measure is not recommended as it does not assess the direction and magnitude of miscalibration, has poor interpretability because it merely produces a p-value, has limited statistical power especially in small samples, and is too sensitive for very large samples. If its use cannot be avoided, it should at least be reported alongside other calibration measures. |
| **Clinical usefulness** (clinically relevant threshold value(s) is required) | Sensitivity  [2, 5, 6, 32, 38, 39, 47, 48] | Proportion of subjects with the outcome correctly identified by the model as such | The higher the value (closer to 100%), the less likely it is that the model will miss subjects with the outcome (less false-negatives). |
|  | Specificity  [2, 5, 6, 32, 38, 39, 47, 48] | Proportion of subjects without the outcome correctly identified by the model as such | The higher the value (closer to 100%), the less likely the model returns false-positive results. |
|  | Accuracy  [5, 8, 37, 48] | Proportion of subjects correctly classified | The closer the value is to 100%, the better the model predictions.  **Caveat:** Accuracy ignores clinical context (relative weights of benefits and harms). It is recommended instead to use more informative summary measures, such as the Net Benefit. |
|  | Net Benefit (NB) and Decision curve analysis (DCA)  [2, 4, 6, 28-30, 32, 35, 39-41, 49, 50] | NB is the benefit of true positive classifications penalized for the harm of false positive classifications. DCA examines the Net Benefit over a range of thresholds. | NB > 0 indicates that the model does more good than harm. |
|  | Relative utility and relative utility curve  [2, 6, 28, 40] | Relative utility is the maximum predicted utility (net benefit) of the model divided by the predicted utility of perfect prediction | Relative utility ranges from 0 (chance prediction) to 1 (perfect prediction). Similar to DCA, a relative utility curve can be obtained, which plots relative utility against a range of thresholds. |
| **Reclassification** (clinically relevant threshold value(s) is required) | Reclassification table  [4, 6, 16, 28, 33, 35] | Compares classifications based on a model and another version of the model (e.g., with an additional marker) | Deviation in counts from predefined risk categories indicate the impact of changes to the model.  **Caveat:** This measure is affected by the cut-offs used to create the risk categories. |
|  | Net reclassification index (NRI)  [2, 4, 6, 16, 22, 27, 28, 30, 32-34, 37, 51, 52] | Net proportion of reclassifications in the correct direction | The larger the value, the larger the positive contribution of the changes to the model.  **Caveat**: The overall NRI is problematic and is not recommended for use [6, 52]. It produces spurious results in the presence of miscalibration [32]. It also does not incorporate clinical consequences and thus needs to be considered separately for events and non-events, reflecting improvements in sensitivity and specificity, respectively. Alternatively, the Net Benefit or the weighted NRI can be used [6]. |
|  | Weighted net reclassification index (weighted NRI)  [6, 16, 22, 52] | Similar to the NRI, except weights are added to accommodate clinical context | Similar interpretation to the NRI, except the weights depend on clinical consequences or cost considerations rather than outcome prevalence.  Note: This measure relates to the Net Benefit (NB) as follows: weighted NRI = ∆NB/ p_t_, where p_t_ is the threshold [6]. |
|  | Integrated Discrimination Index (IDI)  [2, 4, 6, 16, 22, 27, 28, 32-34, 52] | An extension of the NRI over all possible thresholds (i.e., no cut-offs) | The larger the value, the larger the difference is between the two models.  Note: This relates to discrimination slopes between two models [33] and is also equivalent to the difference in Pearson R^2^ [6].  **Caveat**: As with the overall NRI, this measure produces spurious results in the presence of miscalibration [32]. |
| Other measures that were mentioned in the guidance papers are: Concordance K statistic [3], E_average_ (Average absolute difference between observed and predicted risks) [5], Index of predictive accuracy [37], Integrated Calibration Index (ICI) [40], Likelihood ratio test [53, 54], Lorenz curve [8], Loss functions [8], Polytomous discrimination index [14], Positive and negative likelihood ratios [2], Positive and negative predictive values [2, 8, 32, 48], PSEP (a difference in predicted risk) [1], Reclassification calibration [4], Royston and Sauerbrei’s R^2^ [3], and Youden index [4]. | | | |

1. Altman, D.G. and P. Royston, *What do we mean by validating a prognostic model?* Statistics in medicine, 2000. **19**(4): p. 453-473.

2. Cowley, L.E., et al., *Methodological standards for the development and evaluation of clinical prediction rules: a review of the literature.* Diagnostic and Prognostic Research, 2019. **3**: p. 16.

3. Royston, P. and D.G. Altman, *External validation of a Cox prognostic model: principles and methods.* BMC Med Res Methodol, 2013. **13**: p. 33.

4. Steyerberg, E.W., et al., *Assessing the performance of prediction models: a framework for traditional and novel measures.* Epidemiology, 2010. **21**(1): p. 128-38.

5. Vergouwe, Y., et al., *Validity of prediction models: when is a model clinically useful?* Semin Urol Oncol, 2002. **20**(2): p. 96-107.

6. Steyerberg, E.W., et al., *Assessing the incremental value of diagnostic and prognostic markers: a review and illustration.* Eur J Clin Invest, 2012. **42**(2): p. 216-28.

7. Boulesteix, A.L. and W. Sauerbrei, *Added predictive value of high-throughput molecular data to clinical data and its validation.* Brief Bioinform, 2011. **12**(3): p. 215-29.

8. Gail, M.H. and R.M. Pfeiffer, *On criteria for evaluating models of absolute risk.* Biostatistics, 2005. **6**(2): p. 227-39.

9. Steyerberg, E.W., et al., *Validation and updating of predictive logistic regression models: a study on sample size and shrinkage.* Stat Med, 2004. **23**(16): p. 2567-86.

10. Debray, T.P., et al., *Meta-analysis and aggregation of multiple published prediction models.* Stat Med, 2014. **33**(14): p. 2341-62.

11. Debray, T.P., et al., *Aggregating published prediction models with individual participant data: a comparison of different approaches.* Stat Med, 2012. **31**(23): p. 2697-712.

12. Ramspek, C.L., et al., *External validation of prognostic models: what, why, how, when and where?* Clin Kidney J, 2021. **14**(1): p. 49-58.

13. Schnellinger, E.M., W. Yang, and S.E. Kimmel, *Comparison of dynamic updating strategies for clinical prediction models.* Diagn Progn Res, 2021. **5**(1): p. 20.

14. Van Calster, B., et al., *Validation and updating of risk models based on multinomial logistic regression.* Diagn Progn Res, 2017. **1**: p. 2.

15. Jenkins, D.A., et al., *Dynamic models to predict health outcomes: current status and methodological challenges.* Diagn Progn Res, 2018. **2**: p. 23.

16. Wood, A.M. and P. Greenland, *Evaluating the prognostic value of new cardiovascular biomarkers.* Dis Markers, 2009. **26**(5-6): p. 199-207.

17. Archer, L., et al., *Minimum sample size for external validation of a clinical prediction model with a continuous outcome.* Stat Med, 2021. **40**(1): p. 133-146.

18. Bleeker, S.E., et al., *External validation is necessary in prediction research: a clinical example.* J Clin Epidemiol, 2003. **56**(9): p. 826-32.

19. Royston, P., *Explained Variation for Survival Models.* The Stata Journal, 2006. **6**(1): p. 83-96.

20. Nagelkerke, N.J.D., *A note on a general definition of the coefficient of determination.* Biometrika, 1991. **78**(3): p. 691-692.

21. Schemper, M. and J. Stare, *Explained variation in survival analysis.* Stat Med, 1996. **15**(19): p. 1999-2012.

22. McGeechan, K., et al., *Assessing new biomarkers and predictive models for use in clinical practice: A clinician's guide.* Archives of Internal Medicine, 2008. **168**(21): p. 2304-2310.

23. Altman, D.G., et al., *Prognosis and prognostic research: validating a prognostic model.* BMJ, 2009. **338**: p. b605.

24. Debray, T.P., et al., *A new framework to enhance the interpretation of external validation studies of clinical prediction models.* J Clin Epidemiol, 2015. **68**(3): p. 279-89.

25. Justice, A.C., K.E. Covinsky, and J.A. Berlin, *Assessing the generalizability of prognostic information.* Ann Intern Med, 1999. **130**(6): p. 515-24.

26. Moons, K.G., et al., *Risk prediction models: II. External validation, model updating, and impact assessment.* Heart, 2012. **98**(9): p. 691-8.

27. Moons, K.G., et al., *Risk prediction models: I. Development, internal validation, and assessing the incremental value of a new (bio)marker.* Heart, 2012. **98**(9): p. 683-90.

28. Rapsomaniki, E., et al., *A framework for quantifying net benefits of alternative prognostic models.* Stat Med, 2012. **31**(2): p. 114-30.

29. Riley, R.D., et al., *External validation of clinical prediction models using big datasets from e-health records or IPD meta-analysis: opportunities and challenges.* BMJ, 2016. **353**: p. i3140.

30. Steyerberg, E.W. and Y. Vergouwe, *Towards better clinical prediction models: seven steps for development and an ABCD for validation.* Eur Heart J, 2014. **35**(29): p. 1925-31.

31. Vickers, A.J. and A.M. Cronin, *Everything you always wanted to know about evaluating prediction models (but were too afraid to ask).* Urology, 2010. **76**(6): p. 1298-301.

32. Wynants, L., G.S. Collins, and B. Van Calster, *Key steps and common pitfalls in developing and validating risk models.* BJOG, 2017. **124**(3): p. 423-432.

33. Pencina, M.J., et al., *Evaluating the added predictive ability of a new marker: from area under the ROC curve to reclassification and beyond.* Stat Med, 2008. **27**(2): p. 157-72; discussion 207-12.

34. Xanthakis, V., et al., *Assessing the incremental predictive performance of novel biomarkers over standard predictors.* Stat Med, 2014. **33**(15): p. 2577-84.

35. Vickers, A.J. and A.M. Cronin, *Traditional statistical methods for evaluating prediction models are uninformative as to clinical value: towards a decision analytic framework.* Seminars in Oncology, 2010. **37**(1): p. 31-8.

36. Austin, P.C., et al., *Geographic and temporal validity of prediction models: different approaches were useful to examine model performance.* J Clin Epidemiol, 2016. **79**: p. 76-85.

37. Kattan, M.W. and T.A. Gerds, *A Framework for the Evaluation of Statistical Prediction Models.* Chest, 2020. **158**(1): p. S29-S38.

38. Van Calster, B., et al., *Calibration: the Achilles heel of predictive analytics.* BMC Med, 2019. **17**(1): p. 230.

39. de Hond, A.A.H., et al., *Guidelines and quality criteria for artificial intelligence-based prediction models in healthcare: a scoping review.* Npj Digital Medicine, 2022. **5**(1): p. 13.

40. Riley, R.D., et al., *Minimum sample size for external validation of a clinical prediction model with a binary outcome.* Stat Med, 2021. **40**(19): p. 4230-4251.

41. Riley, R.D., et al., *Minimum sample size calculations for external validation of a clinical prediction model with a time-to-event outcome.* Stat Med, 2022. **41**(7): p. 1280-1295.

42. Chambless, L.E. and G. Diao, *Estimation of time-dependent area under the ROC curve for long-term risk prediction.* Stat Med, 2006. **25**(20): p. 3474-86.

43. Altman, D.G., *Prognostic models: a methodological framework and review of models for breast cancer.* Cancer investigation, 2009. **27**(3): p. 235-243.

44. Van Calster, B., et al., *ROC curves for clinical prediction models part 3. The ROC plot: a picture that needs a 1000 words.* J Clin Epidemiol, 2020. **126**: p. 220-223.

45. Verbakel, J.Y., et al., *ROC curves for clinical prediction models part 1. ROC plots showed no added value above the AUC when evaluating the performance of clinical prediction models.* J Clin Epidemiol, 2020. **126**: p. 207-216.

46. van Houwelingen, H.C., *Validation, calibration, revision and combination of prognostic survival models.* Stat Med, 2000. **19**(24): p. 3401-15.

47. Reilly, B.M. and A.T. Evans, *Translating clinical research into clinical practice: impact of using prediction rules to make decisions.* Ann Intern Med, 2006. **144**(3): p. 201-9.

48. Dent, T.H., et al., *Risk prediction models: a framework for assessment.* Public Health Genomics, 2012. **15**(2): p. 98-105.

49. Kappen, T.H.v.K., W. A.; van Wolfswinkel, L.; Kalkman, C. J.; Vergouwe, Y.; Moons, K. G. M., *Evaluating the impact of prediction models: lessons learned, challenges, and recommendations.* Diagnostic and Prognostic Research, 2018. **2**: p. 11.

50. Katki, H.A. and I. Bebu, *A simple framework to identify optimal cost-effective risk thresholds for a single screen: Comparison to Decision Curve Analysis.* Journal of the Royal Statistical Society Series A-Statistics in Society, 2021. **184**(3): p. 887-903.

51. Steyerberg, E.W., et al., *Prognosis Research Strategy (PROGRESS) 3: prognostic model research.* PLoS Medicine / Public Library of Science, 2013. **10**(2): p. e1001381.

52. Pencina, M.J., R.B. D'Agostino, Sr., and E.W. Steyerberg, *Extensions of net reclassification improvement calculations to measure usefulness of new biomarkers.* Stat Med, 2011. **30**(1): p. 11-21.

53. Vergouwe, Y., et al., *A closed testing procedure to select an appropriate method for updating prediction models.* Stat Med, 2017. **36**(28): p. 4529-4539.

54. Hlatky, M.A., et al., *Criteria for evaluation of novel markers of cardiovascular risk: a scientific statement from the American Heart Association.* Circulation, 2009. **119**(17): p. 2408-16.
